# Microbiome spatial scaling varies among members, hosts, and environments across model island ecosystems

**DOI:** 10.1093/ismejo/wraf228

**Published:** 2025-10-13

**Authors:** Jason L Baer, Kacie T Kajihara, Leena L Vilonen, Allie J Hall, Cadie M Young, Danyel K Yogi, Matthew C I Medeiros, Anthony S Amend, Nicole A Hynson

**Affiliations:** Pacific Biosciences Research Center, University of Hawai‘i at Mānoa, Honolulu, Hawaiʻi, USA; Pacific Biosciences Research Center, University of Hawai‘i at Mānoa, Honolulu, Hawaiʻi, USA; Hawai‘i Institute of Marine Biology, University of Hawai‘i at Mānoa, Honolulu, Hawaiʻi, USA; School of Global Environmental Sustainability, Colorado State University, Fort Collins, Colorado, USA; Pacific Biosciences Research Center, University of Hawai‘i at Mānoa, Honolulu, Hawaiʻi, USA; Pacific Biosciences Research Center, University of Hawai‘i at Mānoa, Honolulu, Hawaiʻi, USA; Pacific Biosciences Research Center, University of Hawai‘i at Mānoa, Honolulu, Hawaiʻi, USA; Pacific Biosciences Research Center, University of Hawai‘i at Mānoa, Honolulu, Hawaiʻi, USA; Pacific Biosciences Research Center, University of Hawai‘i at Mānoa, Honolulu, Hawaiʻi, USA; Pacific Biosciences Research Center, University of Hawai‘i at Mānoa, Honolulu, Hawaiʻi, USA

**Keywords:** species area relationships, fungi, bacteria, spatial scaling, bromeliad microbiomes, environmental microbiomes, host-associated microbiomes, food webs

## Abstract

The species area relationship is a classic ecological law describing the relationship between habitat increase and the number of species. Species area relationships are resoundingly positive across macrobes such as plants and animals, and emerge through nonexclusive stochastic and deterministic processes including changes in immigration and extinction, drift, and environmental heterogeneity. Due to unique attributes of the microbial lifestyle, they may not abide by similar rules as macrobes, especially when it comes to spatial scaling. We predict that host-associated microbiomes will exhibit shallower species area relationships than free-living microbiomes due to strong host filtering, and that the species area relationships of bacteria will be shallower than fungi due primarily to differences in dispersal ability. We test these predictions in a relatively simple field system where bromeliad phytotelmata comprise aquatic ecosystems that support invertebrates and environmental substrates such as detritus. Larger phytotelmata generate larger habitat islands for microbiomes allowing us to explicitly examine their species area relationships. We find that the species area relationships of free-living and host-associated microbiomes differ, as do those of microbiome members. By assessing the relationship between environmental conditions and richness, and measuring diversity across scales, we posit that these observed differences in species area relationships are owed to differences in realized niches and dispersal abilities among microbes. These findings highlight that the classic laws of biological spatial scaling do not necessarily accurately represent microbiomes, and that the influence of area on diversity appears to be more important for some microbiomes and microbes than others.

## Introduction

Biological spatial scaling, or how area size influences biodiversity, impacts everything from conservation planning to earth systems modeling to the study of biogeography [1, 2]. Of particular consequence is the relationship between the number of species and area, known as the species area relationship (SAR) [1]. Studies have examined SARs across diverse organisms and habitat types inclusive of islands (ISARs), discrete habitat patches, and spatially nested areas [3–5]. These efforts have coalesced upon two main findings: (i) SARs are often well-defined by simple mathematical relationships, such as a power law function between the number of species and area; and (ii) there is resounding evidence for positive SARs across organisms ranging from plants to mammals and other vertebrates to invertebrates [6, 7]. Some of the primary forces shaping this positive correlation between macroorganism species richness and area include environmental heterogeneity, niche partitioning, and dispersal limitation, along with speciation and extinction dynamics [8]. However, due to unique aspects of the microbial lifestyles such as the exchange of mobile genetic elements among microbes that can rescue and potentially homogenize diversity within and among populations, and the apparent lack of dispersal limitation among others [9, 10], it is unlikely that the laws of biological spatial scaling apply equally as well to microbes as they do to macrobes. Given that microbes drive ecosystem processes such as carbon and nutrient cycling and are requisite symbionts for basically all life on our planet, a better understanding of the patterns and underlying processes that govern microbiome spatial scaling is critical.

**Figure 1:**
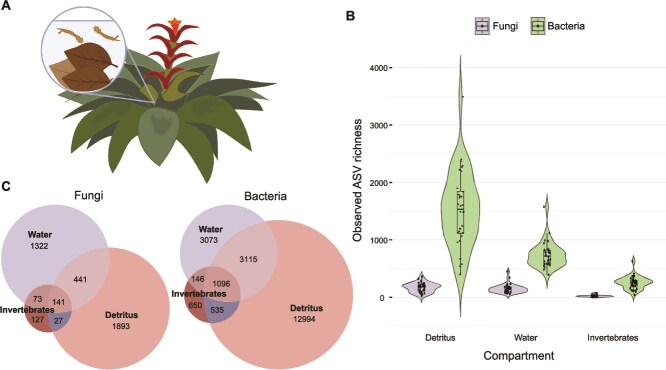
(A) Cartoon depiction of a bromeliad island where phytotelmata capture falling canopy tree leaves and collect rainwater in varying quantities and volumes depending on their size, which then establishes aquatic habitats for hosts such as mosquito larvae. In the current study the relationship between island size and environmental (free-living) or host-associated microbiome richness (fungi and bacteria) was assessed and the mechanisms behind these patterns gleaned from additional measurements of environmental conditions among islands, patterns of microbiome species turnover and by accounting for sampling effects. (B) Violin plots comparing alpha diversity (observed amplicon sequence variant-ASV richness) of each bromeliad island compartment: detritus, water, and invertebrates. Across all bromeliad islands, richness differed significantly among free-living and host-associated bacterial and fungi (*P* < .05). (C) Venn diagrams of fungal and bacterial amplicon sequence variants (ASVs) overlap among island compartments. Bromeliad image in (A) made using Biorender.com.

One of the strongest determinants of microbiome variation is whether they are environmental (free-living), or associated with a host [11]. Whereas hosts generally inherit a significant portion of their microbiome from their environments, it is unknown whether the size of a host’s habitat impacts the richness of its microbiome. The additional filter of host compatibility tends to lead to lower overall microbial richness in hosts relative to environments, but whether host-associated microbiome laws of spatial scaling are similar to those for free-living is yet to be resolved [10, 12, 13]. Also, if factors beyond source-sink dynamics and host filtering differentially influence free-living versus host-associated microbiome spatial scaling, defining these has important implications for microbiome conservation, host performance, and ecosystem services [14]. Furthermore, patterns of spatial scaling among members of the microbiome such as fungi and bacteria may differ and be driven by disparate mechanisms. To discern these patterns and processes requires a natural study system made up of habitat patches that vary in size, while controlling for variation in climate and other landscape-scale abiotic factors known to influence microbiome richness [15]. Bromeliads (*Bromeliaceae*) phytotelmata are a fairly dynamic system that collect rainwater and canopy leaves, which becomes detritus, creating aquatic ecosystems that often house an array of invertebrates (Fig. 1A, [16]). Bromeliads have long been used as a natural model study system of food web ecology [17], but also offer a unique opportunity to study microbiome spatial ecology as each bromeliad represents a habitat island. Here, we leverage a bromeliad garden that contains various bromeliad species which vary in size, and therefore vary in their water and detritus holding capacities, leading to variation in island area for microbiomes and their invertebrate hosts.

Several studies have examined microbial SARs for fungi and bacteria using DNA barcode markers considered to be somewhat equivalent to species (sometimes referred to as Taxa Area Relationships or TARs) and generally find that they are positive [13, 18–21]. However, to date, our understanding of the mechanisms shaping microbial SARs is limited [20, 22, 23]. Whereas there is strong evidence for host and environmental filtering affecting microbial community assembly, it is less clear how these, and other factors such as environmental heterogeneity and dispersal limitation may influence microbiome richness across spatial scales [24]. Fortunately, some mechanisms that underlie microbial SARs can be gleaned from spatially explicit microbiome data while controlling for sampling biases that often plague studies of macrobe SARs [25, 26]. For example, whereas total community census data across islands of various sizes is often out of reach for macrobes, leveraging natural study systems that range in size and contain relatively simple microbiomes such as aquatic habitats like tree holes [18] or bromeliad phytotelmata [27] overcome this “passive sampling” bias. Concurrently, we can assess the influence of ecological factors, such as environmental heterogeneity and dispersal limitation, on microbiome SARs by measuring whether environmental conditions among islands impact species richness and assessing other facets of diversity beyond richness, such as beta diversity and community dispersion, relative to island size.

We ask whether free-living and host-associated microbiomes (in this case communities of bacteria and fungi) within bromeliad phytotelmata island ecosystems inclusive of fresh water, detritus, and invertebrates have positive SARs similar to the vast majority of macrobes, and test whether these SARs differ. We then ask whether the mechanisms underlying microbiome SARs differ between free-living and host-associated microbiomes as well as between fungi and bacteria. We test the following predictions: (i) free-living microbiomes are more diverse and significantly contribute to the composition of host-associated microbiomes, which will be less diverse, (ii) overall SARs will be positive, but host-associated microbiome richness over hosts’ habitat area will be shallower relative to free-living microbiomes and, (iii) along with island size, environmental heterogeneity, and dispersal limitation will differentially influence the richness of free-living and host associated fungi and bacteria. To test the relative contribution of environmental heterogeneity and/or dispersal limitation on free-living and host-associated microbiome richness over area, we model the predictive power of numerous environmental factors and test how microbial species turnover (beta diversity and dispersion) varies across spatial scales. Although disentangling the relative contributions of these mechanisms on SARs is challenging [28], our use of a relatively simple natural study system, where metacommunity dynamics are highly likely due to the close proximity of islands, combined with assessing multiple diversity metrics and measuring specific environmental features known to define the realized niches of our microbiomes [29], allows us to draw strong inferences. Combined, these results will provide new insights into the spatial ecology of microbiomes that will enable better-informed predictions of community assembly rules and how microbial assembly processes may vary across scales, both between environmental versus host-associated microbiomes as well as between fungi and bacteria.

## Materials and methods

### Study system

To measure microbiome SARs among habitat islands of various sizes in a natural setting we sampled bromeliads at the Lyon Arboretum in the upper Mānoa Valley on the island of Oʻahu, Hawaiʻi, USA (21.3330° N, 157.8015° W). Lyon Arboretum has a tropical rainforest climate at an elevation of 151 m with a mean annual rainfall of 4200 mm/year. Lyon contains a bromeliad garden in which plants range ~100-fold in their phytotelmata water holding capacity (tanks ~0.03–3 l) all in close proximity (Supplemental Movie). Bromeliads are not native to Hawaiʻi and assemble relatively simple foodwebs dominated by different trophic guilds of mosquitoes, in particular, their larvae [30]. These attributes make this an ideal study system to assess the relationship between island size and microbiome richness and the mechanisms underlying these patterns. During periods of heavy rainfall bromeliad phytolemata can be “flushed,” potentially leading to secondary succession. To avoid these types of temporal disturbances that could affect microbiome richness or island area, prior to sampling we examined annual daily trends in rainfall over the previous five years, identified timeframes with minimal disturbance where average daily rainfall was consistently under 10 mm and collected all samples over one of these 17-day periods (30 November 2022 and 16 December 2022). Precipitation was consistent throughout our entire study with daily rainfall keeping the bromeliad tank water levels stable (Supplemental Fig. S1; data from Hawaiʻi Climate Data Portal, https://www.hawaii.edu/climate-data-portal/data-portal/). Although some studies of bromeliad phytolemata water chemistry find anoxic conditions selecting for specific microbes, our particular study system is dominantly aerobic likely due to more frequent flushing events (4–5 times a year) and cooler temperatures relative to other study sites in the tropics [31].

### Field methods

We chose 32 bromeliad islands of a few common species in the garden of different sizes resulting in >250*-*fold range in size for detrital habitats and a >90-fold range for aquatic (Supplemental Movie, Supplemental Fig. S2). To account for the potential influence of variation in bromeliad species’ morphology that could possibly impact microbial richness we measured physical characteristics of the bromeliads including: complexity (number of leaves, which is directly proportional to the number of segregated axils containing water), diameter (cm), and height (cm). We measured a range of environmental variables that have previously been shown to significantly impact microbiome diversity and/or community composition [29] including percent carbon and percent nitrogen of detritus, as well as dissolved oxygen, pH, temperature, and nitrate concentrations of the standing bromeliad water. Water variables measured *in situ* included dissolved oxygen and temperature, which were measured with a dissolved oxygen probe (Milwaukee MW600 PRO) and standard digital thermometer. We then collected all of the standing water in each bromeliad phytotelmata using bleach-sterilized plastic pipettes and factory-sterilized serological pipettes. The plastic pipettes had an 8 mm aperture to ensure collection of all invertebrates, the majority of which were the mosquito larvae of *Wyeomyia mitchellii*, a bromeliad specialist, followed by *Aedes albopictus* which are ~6–7 mm in length and ~1 mm in width. Water was stored in autoclaved plastic bottles in a cooler on ice until sampling was completed. All detritus from the bottom of every axil of each bromeliad was collected with sterilized forceps, placed in 50 ml sterile tubes, and stored in a cooler on ice until sampling was completed. The water and detritus were immediately brought back to the laboratory for further processing. Upon returning to the lab, we measured the pH of the water samples (Mettler Toledo laboratory pH sensor). As a measure of island size, we quantified the total volume of water per bromeliad, a metric shown to be an appropriate proxy for aquatic microbes’ habitat area [18, 32, 33].

### Sample preparation

To assess the invertebrate gut microbial community, all water samples were run through a coffee filter to collect the invertebrates from each bromeliad island. Invertebrates were then transferred from the coffee filter to sterile glass petri dishes using a bleach sterilized squirt bottle filled with Milli-Q water. To control for the potential effects of age/stage on gut microbiome diversity we only collected late-instar pupa of mosquitoes (~>6 mm). Total number of late-instar invertebrates was counted in every bromeliad and then each invertebrate was briefly sterilized in 70% ethanol to remove outer microbial communities and rinsed in Milli-Q to remove the ethanol. All invertebrates were frozen at −80°C within 12 h of collection. To preserve DNA for storage, invertebrates were then freeze dried for 24 h. Invertebrate samples were then weighed and stored with desiccant until further processing.

To assess the aquatic microbiome, we filtered the water that passed through the coffee filters through three size class filters (5 μm and 1.2 μm to capture fungi and bacteria, and 0.2 μm to capture any additional bacteria that passed through the previous filters; STERLITECH polyethersulfone membrane filters) using a peristaltic water pump (Masterflex L/S Series) at 100 rpm. The total number of filters per size class varied among bromeliads (Supplemental Table S1). From each bromeliad all 0.2 μm filters were cut into quarters using ethanol-flame sterilized scissors and randomly pooled, then four quarters were placed in 1.5 ml sterile tubes. The 5 μm and 1.2 μm filters were pooled together by bromeliad, with two quadrants of each filter size placed in 1.5 ml tubes for future processing. All tubes were frozen at −80°C within 12 h of collection until further processing. As an additional environmental variable measurement, water collected after 0.2 μm filtration was frozen for nitrate concentration analysis (Biotech Synergy LX Microplate Reader).

To preserve detrital DNA, we froze detritus samples immediately after collection at −80°C and then freeze-dried samples for 24–72 h until completely dry. As an additional measure of island size, the dry weight to the nearest 0.1 mg of the detritus from each bromeliad was measured. Leaf litter or detritus dry weight, in our case, have strong positive correlations with total surface area, but they are not 1:1 due to differences in leaf density [34]. To control for this, our experimental field site consisted of a single small bromeliad garden where the type of canopy leaves contributing to detritus is basically constant across the site. Furthermore, due to the methodological challenges of accurately measuring total surface area of leaf litter or detritus, dry weight has long been used as a proxy by which to compare aspects of microbial ecology, including diversity metrics as we have here [35]. Environmental characterization of the detritus included total carbon and nitrogen analysis, run by the Crow Soil Ecology and Biogeochemistry Lab at the University of Hawaiʻi at Mānoa (https://soilandcarbon.com/services/). Detritus samples were then divided into 250 mg portions for DNA extraction and sequencing.

### DNA extraction and PCR

High-throughput amplicon sequencing of the fungal and bacterial small subunit ribosomal RNA (rRNA) genes (18S and 16S, respectively) was used to assess the diversity and membership of free-living environmental and host-associated microbiomes. We extracted DNA from technical replicates of the most frequent sampling intensity per bromeliad (sample median) for water filters (median = 3) and aliquots of detritus (median = 10) and extracted from all pooled-by-bromeliad island invertebrate samples (one pool per island except for one island that had two pools and one that had zero, *n =* 32). Some bromeliad islands had fewer than the median in which case all samples were extracted and sequenced. For a random subset of bromeliad islands (six total), we extracted DNA from all technical replicate water filters and detritus to assess whether sequencing depth adequately captured bacterial and fungal richness for the subsampled compartments of the remaining islands (see Supplemental Table S1 for the number of tubes extracted per sample). Total sample numbers were 76 for the 0.2 μm filters plus six blanks, 98 for the pooled filters plus 10 pooled blanks, and 223 for the detritus, along with 18 DNA extraction negative controls, resulting in a grand total of 463 samples including the invertebrates. Water filters, detritus, and invertebrates were transferred to MP Bio Lysing Matrix A tubes (MP Biomedicals, OH, USA) and homogenized for 3 min at 1800 rpm on a MP Bio Fast-Prep 96 homogenizer (MP Biomedicals, OH, USA). DNA was extracted from water and detritus samples using the Qiagen MagAttract PowerSoil kit using randomized plate extraction with automatic extraction on a KingFisher automated extraction & purification system (Waltham, MA). Invertebrate DNA was extracted using the Macherey-Nagel NucleoMag tissue kit with the same KingFisher system (Waltham, MA). The only exception to the manufacturer’s instructions was the addition of a ProK digestion to all DNA extractions.

Prior to PCR amplification, we diluted our samples 1:10 in nuclease free water. For any samples that failed to amplify during the PCR, we diluted the samples further to 1:100. A primer test between 18S rRNA genes and the internal transcribed spacer (ITS) region for fungi on a small subset of samples showed that 18S rRNA genes captured a greater phylogenetic diversity of fungi known to live in aquatic environments (ex. chytrids), thus we chose 18S as our target region. We amplified DNA using the KAPA 3G plant protocol kit and DNA marker barcodes specific to each sample (NEXTERA kit; Illumina Inc., CA, USA), using the 515F/806R primer set for bacterial 16S rRNA genes [33] and 18S-82F/Ek-516R for fungal 18S rRNA genes [36, 37]. Fungal and bacterial samples were then sequenced on a MiSeq System (Illumina) with paired-end 300 bp-read sequencing by the Advanced Studies in Genomics, Proteomics and Bioinformatics (ASGPB) at the University of Hawaiʻi at Mānoa (http://www.hawaii.edu/microbiology/asgpb/).

### Bioinformatics

Fungal data were processed in QIIME2 version 2024.2 [26], starting with 15 169 060 raw reads. Sequences were trimmed, denoised, and quality-filtered using the DADA2 plugin in QIIME2 [38], resulting in 9 307 133 reads. Taxonomy was assigned using the classify-consensus-blast algorithm [39] against the SILVA database version 138.2 [40], resulting in 2 922 137 assigned reads, 169 894 unassigned reads and the remainder assigned outside Kingdom fungi. Bacterial data were processed using the MetaFlow|mics pipeline [41] as in [12] starting with 19 861 551 raw reads, 18 495 594 remained after DADA2, 15 850 908 were assigned taxonomy, and 15 847 530 passed through the full pipeline. For both fungi and bacteria, the forward read data were higher quality and resulted in more reads passing quality control, thus we proceeded with forward read data only. A total of 4744 fungal amplicon sequence variants (ASVs) and 27 280 bacterial ASVs passed quality checks. For bacteria, only the 0.2 μm water filter samples were used to assess bacterial diversity in the water, whereas both the 0.2 μm and pooled 5 μm and 1.2 μm filters were used for fungi.

Both datasets were imported into R version 4.2.1 for downstream analysis [42]. Putative contaminants were removed from each taxa table with the decontam package [43], using the “prevalence” method to compare the presence/absence of sequences in DNA extraction and PCR negatives with those in true samples. For water samples, blank filters were also used as negative controls. As an additional quality control measure low-abundance samples and ASVs were culled. Culling thresholds were determined by assessing log-transformed sample and ASV read counts for “breaks” in their plotted distributions (Supplemental Fig. S3). For fungi, samples with 300 or fewer reads and ASVs with three or fewer reads were culled. For bacteria, samples with 2901 or fewer reads and ASVs with four or fewer reads were culled. The resulting datasets consisted of 4024 ASVs and 418 samples for fungi, and 21 609 ASVs and 402 samples for bacteria.

### Sampling sufficiency, overall richness, and composition

To determine whether our microbiome communities were sufficiently sampled, we generated ASV accumulation curves using the iNEXT package [44] by bromeliad compartment (invertebrate, water, and detritus) for each locus (fungi and bacteria) and each replicate sample. All accumulation curves reached an asymptote (Supplemental Figs. S4–S9), so observed ASV richness was used to represent alpha diversity. Richness among compartments and microbes met the assumptions of ANOVA so we used this model along with post hoc Tukey's Honestly Significant Difference (HSD) tests to assess differences in overall and pairwise comparisons of richness among bacteria and fungi. Overlap in ASV presence among compartments within a locus was visualized using the eulerr package [45]. To visualize the community composition of each bromeliad island within a compartment and locus, we generated relative abundance bar plots in ggplot2 [46]. To assess nestedness of fungal and bacterial communities among bromeliad compartments (water, detritus, and invertebrates) we constructed presence-absence matrices by pooling all samples within each compartment across bromeliads and taking the union of observed ASVs. Nestedness was quantified using the NODF metric (vegan::nestednodf) and compared against 1000 quasiswap null matrices generated with oecosimu, which preserve both row and column totals.

### Building species area relationships

To determine the species area relationships (SARs) for free-living and host-associated fungi and bacteria, we ran linear regressions separately by bromeliad compartment with richness as the response variable. The “island size” predictor variables were water volume (ml) for aquatic fungi and bacteria, detritus weight (mg) for detrital fungi and bacteria, and both water volume and detritus weight for invertebrate fungi and bacteria. Richness and island size were log-transformed in all regressions. We used the statistical significance of the predictor (*P-*value; α = .05) and *R*^2^ (amount of variance explained by the predictor) to evaluate the strength of the predictor in the linear model. z-scores (slopes) were calculated for each SAR that was statistically different from a slope of zero. Higher z-scores are generally an indicator of higher dispersal limitation [7, 13]. The slopes of each regression were compared by fungi or bacteria across compartments, and within compartments between microbiome members using pairwise t-tests with the Benjamini-Hochberg method [47] and the multtest package [48] to correct for multiple comparisons. Bootstrapped slope distributions for the t-tests were generated by randomly pairing richness and area from each original dataset 1000 times with replacement [13].

### Assessment of environmental heterogeneity and microbial dispersal effects on species area relationships

Mechanisms underlying statistically significant SARs can be owed simply to passive sampling bias. Because our microbial species accumulation curves by each technical replicate (sample) saturated (i.e. we achieved a total census of bacteria and fungi among each bromeliad compartment; Supplemental Figs. S4–S9), this negated any further tests of passive sampling bias. However, disproportional effects, or changes in community evenness related to island size, are an important consideration for interpreting SARs. As a measure of community evenness, for each compartment and for fungi and bacteria, we calculated Hill number *q* = 1. *Q* = 1 was chosen as the most appropriate measure of community evenness as it accounts for both rare and common species and normalizes for differences in species richness, allowing for robust comparisons among islands [49]. We then regressed *q* = 1 against the log-transformed island size (measured as detritus weight or water volume) for fungi and bacteria in each compartment. Linear regression was used to test whether evenness scaled significantly with island size, and the coefficients of determinations (*R*^2^) were used to quantify the proportion of variance in evenness explained by island size. If the relationship between *q* = 1 and island size is statistically significant, then disproportionate effects likely influence the SAR. For example, a negative correlation between *q* = 1 and island area would indicate that on larger islands communities are becoming less even.

To assess whether environmental heterogeneity or host number and/or weight affects microbiome richness, we ran multiple linear regression with environmental and physical variables related to bromeliad area and hosts as predictors in our models. Variables common to all analyses included bromeliad diameter, height, complexity (number of leaves per bromeliad), invertebrate number, and invertebrate weight. Detritus-specific variables included percent carbon and percent nitrogen. Water-specific variables included dissolved oxygen, pH, temperature, and nitrate concentration. All variables were added, and the stepAIC function from the MASS package [50] was used to select the final model for each compartment within a locus. The relative importance of each variable in the full model was assessed with the relaimpo package [51].

To assess how dispersal limitation or environmental filtering may affect SARs, we measured turnover in community composition for fungi and bacteria in each compartment, both within and among bromeliad islands. Assuming that SARs are statistically significant, if environmental conditions do not explain a significant portion of the variation in microbiome member richness over area, or only explain a marginal proportion of it, dispersal limitation or stochastic processes such as drift may be the primary mechanisms underlying significant SARs [52]. For example, if there is an overall positive relationship between beta diversity and island size that cannot be attributed to environmental heterogeneity alone, this may be indicative of dispersal limitation asymmetrically contributing to richness on larger islands. If indeed dispersal limitation is more likely on larger islands, then we should detect greater beta dispersion within larger bromeliads. Beta diversity was calculated using Bray–Curtis dissimilarities in vegan [53]. Among bromeliad islands, community composition distance matrices over island size (water volume or detritus weight) were compared using a Mantel test with Spearman’s correlations and 9999 iterations. Linear regressions between changes in community composition and island size (milligrams detritus or milliliters water) were performed by microbe and compartment, and slopes were bootstrapped using the method described above to generate distributions for pairwise t-tests. Within bromeliad islands, beta dispersion was calculated by sample for free-living microbiomes as Bray–Curtis distances from their centroids and values for fungi or bacteria were regressed over the log of island size. We then used linear regression to test for slopes that differed from zero and examined the coefficient of determination (*R*^2^) as an indicator for the amount of variation in beta dispersion that was explained by island size. Concurrently, we calculated Raup Crick Bray as in [54] to preserve the relative abundance of bacterial or fungal taxa. The benefit of this approach as opposed to simply observed betadiversity is that it allows for the comparison of observed species turnover to a null distribution, where both distributions are normalized to values between −1 and 1. Significant positive departures from the null (> 0.95) indicates factors such as environmental filtering or dispersal limitation along with drift strongly affect community turnover, whereas significantly negative values (<−0.95) represent the potential impact of homogenizing dispersal on betadiversity. No differences in observed community turnover from the null would be indicative of ecological drift [55]. For all statistical analyses an α ≤ 0.05 was considered statistically significant.

## Results

### Diversity and nestedness

Overall fungal richness of detritus was not significantly different from water, but both were significantly richer than in invertebrates (Fig. 1B, Supplemental Table S2). For bacteria, detritus was richer than water and, similar to fungi, both were significantly more rich than in invertebrates (Fig. 1B, Supplemental Table S2). Excluding unclassified fungi, the most abundant fungal groups by bromeliad compartment were *Capnodiales* and *Hypocreales* in detritus, *Xylariales* and *Eurotiales* in water, and *Sordariales* and *Pleosporales* in invertebrates (Supplemental Fig. S10). For the bacteria, the most abundant groups were within *Gammaproteobacteria* and *Alphaproteobacteria* in detritus and water, and *Alphaproteobacteria* and *Clostridia* in invertebrates (Supplemental Fig. S11). Compositionally across all bromeliad islands, nearly two-thirds of free-living fungi were also found in hosts (65.6%), whereas free-living bacteria accounted for nearly three-quarters of those detected in hosts (73.2%). Based on nestedness quantification via NODF, the temperatures of bacterial and fungal communities across compartments indicated strong nestedness, especially for bacteria, and null modelling revealed that these patterns were significantly different from random (Supplemental Table S3; Supplemental Fig. S12).

### Species-area relationships

**Figure 2:**
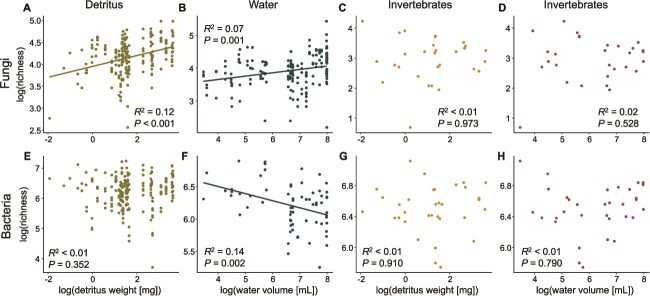
Species-area relationships (SARs) between microbial amplicon sequence variant (ASV) richness (y-axis) and measures of bromeliad island size (x-axis; total detritus weight in mg or total water volume in ml) for fungi (A–D) and bacteria (E–H). Both axes were log-transformed, and relationships were assessed across detritus (A and E), water (B and F), and invertebrate samples (C–D and G–H). Regression lines are shown for loci (fungal and bacterial SSU) and compartments (detritus, water, invertebrates) that had significant relationships (*P* ≤ .05). Additional regression values are listed in Table 1.

We found that host-associated bacterial and fungal richness had no relationship with island size, whether considering hosts in aquatic or detrital habitats and this finding was not confounded by either host weight nor count (Fig. 2, Table 1). Conversely, free-living fungi followed the predicted pattern of positive SARs in detrital and aquatic habitats, whereas free-living bacteria either had no relationship (detritus) or a negative SAR in the case of aquatic bacteria (Fig. 2, Table 1). Specifically, detrital fungi exhibited a significant positive relationship between richness and island size as determined by detritus weight and bromeliad diameter, but detritus weight only explained ~12% of the increase in richness across islands (*P* < .001, *R*^2^ = 0.12; Fig. 2, Table 1), and diameter explained 3.7% (Table 2, Supplemental Table S4). Aquatic fungi also displayed a significant positive relationship between richness and island size as water volume, but again island size only explained minimal (~10%) of the variation in richness across islands (*P* = .001, *R*^2^ = 0.07; Fig. 2; Table 1). As island size increased, detrital fungal communities became significantly more even (*P* < .001, *R*^2^ = 0.17), whereas free-living aquatic fungi showed no change in community evenness with island size (Supplemental Fig. S13, Supplemental Table S5).

**Table 1:** Linear regression values for species area relationships (SARs) between microbiome richness and bromeliad island size (both log-transformed). *P*-values were corrected with the Benjamini & Hochberg control of the false discovery rate [45], and significant regressions have *P*-values in bold (*P* ≤ .05).

Microbe	Compartment	Island Size	*R* ^2^	Adjusted *P*-value	Slope	Intercept
Fungi	Detritus	Detritus weight	0.12	**<.001**	0.12	3.95
	Water	Water volume	0.07	**.001**	0.10	3.25
	Invertebrates	Detritus weight	<0.01	.973	<0.01	2.96
	Invertebrates	Water volume	0.07	.528	0.07	2.55
Bacteria	Detritus	Detritus weight	<0.01	.352	0.03	6.18
	Water	Water volume	0.14	**.002**	−0.11	6.94
	Invertebrates	Detritus weight	<0.01	.910	<0.01	6.50
	Invertebrates	Water volume	<0.01	.790	0.01	6.43

**Table 2:** Summaries of AIC-selected multiple regression models with log-transformed microbial richness as the response variable, and all measured environmental and physical variables as predictors with total detritus weight (A) or total water volume (B). Regressions were run by locus (fungal and bacterial SSU) and bromeliad compartment (detritus (A), water (B), and invertebrates (A and B)). Table values are model coefficients unless otherwise indicated. Asterisks indicate significance (^*^P ≤ .05, ^**^P ≤ .01; ^***^P ≤ .001), and no asterisks indicate *P* > .05. Complexity refers to the number of leaves per bromeliad, and percent carbon and nitrogen were measured of the detritus. Dissolved oxygen, pH, temperature, and nitrate concentration were measured of the bromeliad water.

(a)											
		**Dependent variable: log(richness)**									
		log(invertebrate_wt)	Diameter	Height	Complexity	Invertebrate number	Percent carbon	Constant	*R* ^2^	Residual Std. Error	
Fungi	Detritus		0.004^***^				−0.014	4.527^***^	0.072	0.390 (df = 193)	
	Invertebrates							2.988^***^	0	0.721 (df = 27)	
Bacteria	Detritus			0.005^**^	−0.006	0.002^*^		6.007^***^	0.079	0.518 (df = 192)	
	Invertebrates							6.505^***^	0		
**(b)**											
		**Dependent variable: log(richness)**									
		**log(invertebrate_wt)**	**Diameter**	**Height**	**Complexity**	**pH**	**Temperature**	**Nitrate water**	**Constant**	***R* ^2^**	**Residual Std. Error**
Fungi	Water					−0.089^***^	−0.083^***^	−4.950^*^	6.373^***^	0.238	0.437 (df = 144)
	Invertebrates	0.183^***^			−0.011	0.243^***^			1.518^**^	0.500	0.416 (df = 22)
Bacteria	Water		−0.006^*^	0.005	−0.007	0.068^*^			6.041^***^	0.236	0.303 (df = 56)
	Invertebrates		0.004			0.060			5.851^***^	0.109	

Unlike fungi, free-living detrital bacteria did not have a significant relationship between richness and detritus weight (*P* = 0.352, *R*^2^ < 0.01, Fig. 2; Table 1), but did have a significant relationship with bromeliad height, another metric of island size (Table 2, Supplemental Table S3). However, height only explained 2.8% of the variation in detrital bacterial richness (Table 2, Supplemental Table S4). Free-living aquatic bacterial SARs were significant (*P* = .002; *R*^2^ = 0.14), though with a negative relationship (Fig. 2; Table 1). Bromeliad diameter, another measure of island size, was also a significant predictor of aquatic bacterial richness, explaining 7% of the variation over island size (Table 2, Supplemental Table S4). Host-associated bacterial communities became significantly more even as island size increased whether considering aquatic (*P* = .001; *R*^2^ = 0.33) or detrital (*P* < .001; *R*^2^ = 0.43) island size, whereas aquatic bacterial communities became less even as island size increased (*P* = .001, *R*^2^ = 0.07) and detrital bacteria showed no change in community evenness with island size (Supplemental Fig. S13, Supplemental Table S5).

The Z-score of free-living detrital fungi was highest (0.124), followed by aquatic fungi (0.102), followed by aquatic bacteria (−0.110, Supplemental Fig. S14). When comparing the slopes of our SARs within microbiome members and across compartments, all comparisons were significant (Table 3). When comparing the slopes of our SARs within compartment, but between members (e.g. detrital fungi vs. detrital bacteria), all comparisons were significant except for host-associated bacteria and fungi over detritus weight (Supplemental Table S6).

**Table 3:** Differences in bootstrapped species area relationship (SAR) slope distributions of bromeliad island compartments (detritus, water, and invertebrates) within microbiome members (fungi or bacteria). Slopes were derived from the linear regression between log-transformed microbial richness and log-transformed bromeliad island size (detritus weight or water volume). Bootstrapped distributions were generated by randomly pairing richness and volume 1000 times with replacement. Invertebrate microbial richness was regressed against both detritus weight and water volume, so the specific volume metric is in parentheses. Pairwise Welch’s t-tests were performed within microbes and *P-*values were corrected with the Benjamini & Hochberg control of the false discovery rate [47]. All comparisons were significant (*P* < .001).

Microbe	Compartment 1	Compartment 2	*t*	df	Adjusted *P-*value
Fungi	Detritus	Invertebrates (Detritus weight)	38.17	1118.27	<.001
	Detritus	Invertebrates (Water volume)	14.62	1065.30	<.001
	Detritus	Water	19.01	1961.98	<.001
	Invertebrates (Detritus weight)	Invertebrates (Water volume)	−11.56	1846.31	<.001
	Invertebrates (Detritus weight)	Water	−31.04	1155.26	<.001
	Invertebrates (Water volume)	Water	−9.39	1086.01	<.001
Bacteria	Detritus	Invertebrates (Detritus weight)	19.59	1976.70	<.001
	Detritus	Invertebrates (Water volume)	9.10	1838.71	<.001
	Detritus	Water	102.99	1973.35	<.001
	Invertebrates (Detritus weight)	Invertebrates (Water volume)	−6.80	1738.52	<.001
	Invertebrates (Detritus weight)	Water	88.06	1997.87	<.001
	Invertebrates (Water volume)	Water	75.69	1730.52	<.001

### Environmental predictors of microbiome richness

Overall, the measured environmental characteristics were not significantly correlated with microbiome richness (Table 2). The main exception was free-living aquatic fungi whose richness was significantly related to changes in pH, temperature, and nitrate concentrations among islands (Table 2). These variables alone explained >25% of the increase in fungal richness among islands (Supplemental Table S4). Aquatic bacterial richness and host-associated fungal richness were associated with changes in pH explaining ~7% and 19.5% of the variation in richness among islands, respectively (Table 2 & Supplemental Table S4). Host-associated bacterial richness was not related to the number of invertebrate hosts or their weight, nor were host-associated fungi when considering detrital island size, whereas host-associated fungal richness when considering their aquatic island size was significantly correlated with total invertebrate weight (Supplemental Table S4).

### Beta diversity and dispersion

**Figure 3:**
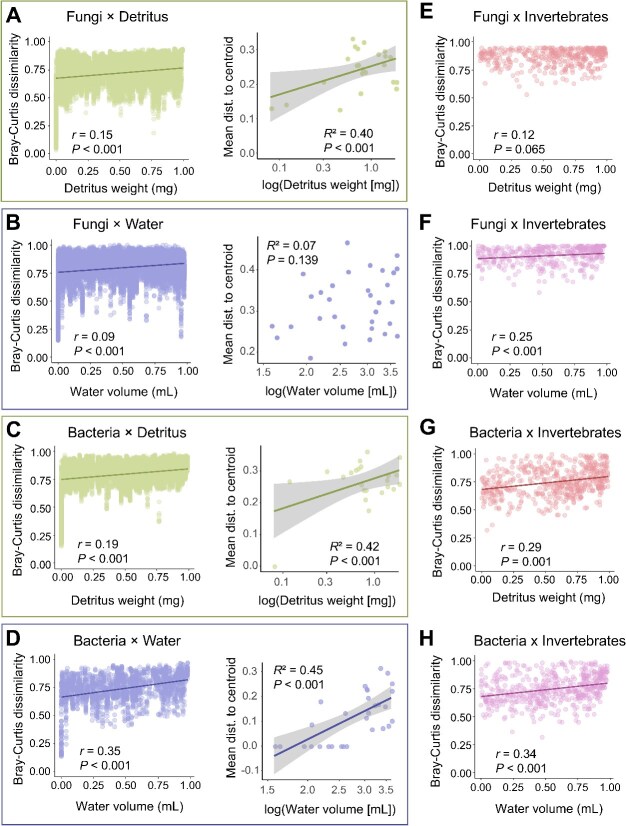
Mantel correlations of Bray–Curtis dissimilarity of community composition over bromeliad island size (total detritus weight in mg or total water volume in ml) for fungi (A, B, E, and F) and bacteria (C, D, G, and H). Correlations were assessed across detritus (A and C), water (B and D), and invertebrate (E–F and G–H) samples. Linear trendlines are displayed for significant relationships (*P* ≤ .05) as a visual aide. Neighboring plots to Mantel correlations reflect the beta dispersion (mean distance to centroid) of free-living (environmental) microbiomes by island size (either detritus weight in mg or water volume in ml).

Except for host-associated fungi when considering their detrital island size, free-living and host-associated microbial species turnover in relation to island size was significant (*P* < .003) and positive across bromeliad islands, with island size explaining a marginal (0.8% for aquatic fungi) to relatively larger proportions of the variation in community composition (12.5% for host-associated bacteria in aquatic islands; Fig. 3A). In general, island size explained more of the variation in free-living and host-associated bacterial community composition than fungal (*r* = 0.19–0.35 versus *r* = 0.09–0.25, respectively, Fig. 3A), and more of the variation among host-associated microbiomes than free-living (*r* = 0.25–0.34 versus *r* = 0.09–0.35, respectively, Fig. 3A). The community turnover rates were significantly different from each other among compartments for bacteria and fungi (*P* < .001; Supplemental Table S7), as well as between fungi and bacteria within the same compartments, with detrital fungi and bacteria having the greatest turnover rates, (*P* < .001; Supplemental Table S8). Overall, host-associated fungal communities were highly dissimilar to each other with often almost complete community turnover regardless of island size (Fig. 3). To examine differences in free-living microbiome community composition as it relates to island size we calculated beta dispersion, or the distance of a community from their centroid in multivariate space, for each bromeliad island and its aquatic or detrital bacterial and fungal communities. We found that community dispersion increased as a function of island size for detrital fungi (*P* < .001, *R*^2^ = 0.40) and bacteria (*P* < .001, *R*^2^ = 0.42) as well as aquatic bacteria (*P* < .001, *R*^2^ = 0.45), but not aquatic fungi (Fig. 3A–D). Raup Crick Bray values for fungi and bacteria among detrital, aquatic, and invertebrate communities were all strongly indicative of environmental filtering, or other processes such as biotic interactions or dispersal limitation along with drift leading to turnover in community membership (≥99% of pairwise comparisons within free-living or host associated fungi or bacteria had Raup Crick BC values >0.95; Supplemental Table S9).

## Discussion

Similar to prior studies, and in support of our first prediction we found that environmental microbiomes are more diverse than host microbiomes, and that fungal and bacterial communities are nested within ecosystem compartments [11, 12, 56]. However, our results of disparate SARs among bromeliad island compartments and microbiome members do not support our hypothesis that source-sink dynamics are the underlying mechanism that determines microbiome richness across scales. Instead, these appear to be microbial Domain- and free-living or host-associated specific [21]. Even though we did find some support for our prediction of positive SARs among specific portions of the microbiome, such as free-living fungi, this pattern did not hold for host-associated fungi nor bacteria regardless of whether they are free-living or host-associated. Instead, host-associated microbiome richness remained relatively constant as their habitat size increased. Overall we found little evidence for the absence of significant SARs among host microbiomes being owed to any sampling biases such as differences in invertebrate numbers or weight among samples. The one exception may be fungal richness when considering their aquatic island size, which was significantly correlated with total invertebrate weight. These results indicate that instead, host physiology significantly modulates microbiome richness [57–59]. However, microbial species turnover, even among small islands, was generally higher for host-associated microbes relative to free-living and highest for host-associated fungi. So, whereas host physiology may limit the number of microbes that occupy a host, the membership of these communities among habitat islands, even of similar size, are vastly different, especially for fungi. This result lends credence to the potential for high functional redundancy among host-associated microbiomes [60]. Alternatively, portfolio effects could be at play, where a subset of specific and consistent members of the core microbiome elicit critical host functions, and other members just represent more transient symbionts [61].

The positive SARs among free-living fungi, although shallower than most macroorganisms [13, 18, 62], were the most comparable to prior studies, indicating that for some free-living microeukaryotes, biological spatial scaling may be constrained by similar factors as plants and animals [19, 22]. Specifically, for free-living detrital fungi, island size and species turnover were more tightly correlated than for free-living aquatic fungi. This, along with our results that: (i) detrital fungal communities have, on average, relatively high beta diversity, (ii) beta dispersion is significantly higher within larger bromeliad islands than smaller ones, and (iii) the z-score for detrital fungi is higher than other members of the microbiome, all strongly indicate that dispersal limitation likely modifies spatial scaling within this group [21]. Although we found little evidence that environmental conditions impact detrital fungal richness, additional unmeasured environmental factors may play important roles determining community dynamics of this group among islands. Indeed, our finding that fungal communities in larger detrital islands were more even hints at the potential for environmental filtering affecting community structure and deserves further study. Conversely, environmental filtering appears to be a critical predictor of free-living aquatic fungal richness. Dispersal limitation does not appear to be a significant factor structuring aquatic fungal communities due to the relatively weak relationship between beta diversity and island size, and no evidence of increasing beta dispersion as a function of island size. Instead, environmental conditions such as water pH, temperature, and nitrate concentration were the best predictors of aquatic fungal richness as island size increased. Furthermore, as island size increased pH significantly decreased (Supplemental Fig. S15A), changes in island size and associated changes in environmental conditions appear to significantly impact aquatic fungal richness in concert. These findings are similar to those of Zhou *et al.* (2024 [5];) where changes in soil pH strongly influenced bacterial and fungal richness across islands of various sizes. As aquatic island size in our study system increased there was a corresponding increase in the ratio of detritus to water, which we infer led to lower pH values in larger islands (Supplemental Fig. S15B [63]). In our case, greater fungal richness on larger islands could be owed to greater environmental niche diversity or homogenous environmental conditions on larger islands that are more conducive for a range of fungi. An important additional consideration is biotic interactions, which we discuss further in the next section.

Free-living bacterial richness had a negative relationship with aquatic island size and no relationship with detrital island size. Whereas negative SARs are uncommon in the literature, previous studies have found this pattern and attribute it to a random distribution of inhabitable patches across landscapes and nutrient availability [22, 64]. In our study system we sampled bromeliad islands of differing sizes, so the spatial proximity of patches may indeed be random. However, an additional explanation may be relatively stronger environmental filtering of aquatic bacteria on larger islands than smaller. Our model incorporating environmental conditions among aquatic islands found that differences in pH explained a significant proportion of the variation in free-living bacterial richness. Because pH was significantly negatively correlated with island size; it is possible that larger islands were only tolerable for specific bacteria [21, 31, 65], despite the relatively high turnover in bacterial community members as island size increased (Fig. 2, *P* < .001, *r* = 0.35). Beta dispersion of aquatic bacterial communities was significantly higher within larger aquatic islands, meaning bacterial communities within these islands were less similar than communities within smaller islands. Larger islands also trended toward less even bacterial communities than smaller islands. We interpret these findings as indication that environmental conditions, such as low pH, strongly filters the number of bacteria that can subsist in larger islands, and that the environmental conditions may be more heterogeneous within larger islands. Unlike prior studies though, we found no evidence that nutrient availability (specifically nitrogen) could be limiting bacterial richness on larger versus smaller islands [66]. The opposing shapes of free-living aquatic fungal SARs versus bacterial and the fact that pH was a significant predictor of changes in richness for both groups provides intriguing evidence for the possibility of biotic interactions among bacteria and fungi shaping their spatial scaling rules. For example, a greater diversity of aquatic fungi may be capable of persisting in the low pH environments found in larger islands due to unoccupied niche space by aquatic bacteria. In our aquatic habitats neither fungal nor bacterial richness was affected by available oxygen, which also did not correlate with aquatic island size, indicating that total water volume is an appropriate measure of total habitat area for these microbes (i.e. communities are not stratified based on anoxic or oxic conditions; Supplemental Figs. S16 and S17).

Although the richness of free-living detrital bacteria was not correlated with island size measured as detrital weight, it was significantly and positively correlated with bromeliad height, which could be considered an alternative metric of island size. Even though for this group the trend between beta diversity and island size was only weakly positive, there was a strong positive correlation between beta dispersion and island size. One interpretation of this result is that environmental filtering may be affecting community membership more within larger islands than smaller, but overall communities are not dispersal limited. An additional consideration is temporal environmental variation such as the potentially fluctuating availability of oxygen that may be strong selective pressures both on richness and community membership, but to test this would require additional longitudinal studies. So, whereas Raup Crick Bray values approached one for all free-living microbes, our additional measures of environmental filtering and dispersal limitation indicate that depending on the group of microbes and their habitat, the impact of these forces on community assembly and turnover likely vary. An important caveat to interpreting SARs is that microbes’ total habitat area is exceedingly difficult to quantify. For example in the case of our study, many bacteria and fungi were found to co-occur in both aquatic and detrital habitats, but without strain resolved data, or more detailed information on these “generalists” realized niches, it is challenging to say whether we have captured the entirety of their habitat area.

In summary, the factors influencing biological scaling differ among members of the microbiome as well as between free-living and host-associated and microbes do not always emulate the positive SARs commonly found among macrobes. For example, whereas free-living aquatic fungi exhibited a positive SAR likely due to environmental heterogeneity and filtering, free-living detrital fungi’s spatial scaling patterns appear to be driven more by dispersal limitation. SARs of free-living bacteria in our study system were either the opposite of fungi or nonexistent. Similarly, host-associated fungal and bacterial richness showed no relationship to island size. These results encourage a reexamination of the universality of positive SARs and provide new information on how microbial community assembly rules vary among free-living and host-associated members of the microbiome as well as between bacteria and fungi.

## Supplementary Material

Species_Area_Supplement_092625_clean_wraf228

## Data Availability

The raw sequence reads generated in this study have been deposited in the NCBI Sequence Read Archive (SRA) under BioProject ID PRJNA1274038 and BioSample Accession numbers SAMN48972843 and SAMN48972844. Associated metadata, including sample collection dates and environmental context, are available via Zenodo at doi:10.5281/zenodo.15670682. All relevant analysis scripts and bioinformatics workflows are available upon request and will be made publicly accessible upon publication.
