# Supplementary material for: Microbiome spatial scaling varies among members, hosts, and environments across model island ecosystems
Source: ISME J. 2025 Oct 13;19(1):wraf228. doi: 10.1093/ismejo/wraf228 (PMC12596279; doi:10.1093/ismejo/wraf228)
Supplement: Species_Area_Supplement_092625_clean_wraf228 [file species_area_supplement_092625_clean_wraf228.pdf]

Supplementary Material for *Microbiome spatial scaling varies among members, hosts, and environments across model island ecosystems*

Authors Jason L. Baer[1], Kacie T. Kajihara[1,2], Leena L. Vilonen[3], Allie J. Hall[1], Cadie M. Young[1], Danyel K. Yogi[1], Matthew C.I. Medeiros[1], Anthony S. Amend[1], & Nicole A. Hynson^[1]
[1]Pacific Biosciences Research Center, University of Hawaiʻi at Mānoa
[2]Hawaiʻi Institute of Marine Biology, University of Hawaiʻi at Mānoa
[3]School of Global Environmental Sustainability, Colorado State University

Supplemental Movie: Movie showing the sampling site for this study, the bromeliad garden at Lyon Arboretum on the island of Oʻahu in Hawaiʻi, USA (21.3330° N, 157.8015° W). Bromeliad phytotelmata varied in size generating island habitats of water and detritus of various areas, but all growing in close proximity.
https://www.dropbox.com/scl/fi/vt235e5ktsvail2ktuhz5/IMG_8918.MOV?rlkey=86s4f12p1y57scisui2vavoyu&dl=0

Figure S1. Rainfall levels across our sampling period at the Lyon Arboretum on the island of Oʻahu in Hawaiʻi, USA (21.3330° N, 157.8015° W). Data are from the Hawaiʻi Climate Data Portal (https://www.hawaii.edu/climate-data-portal/data-portal/).

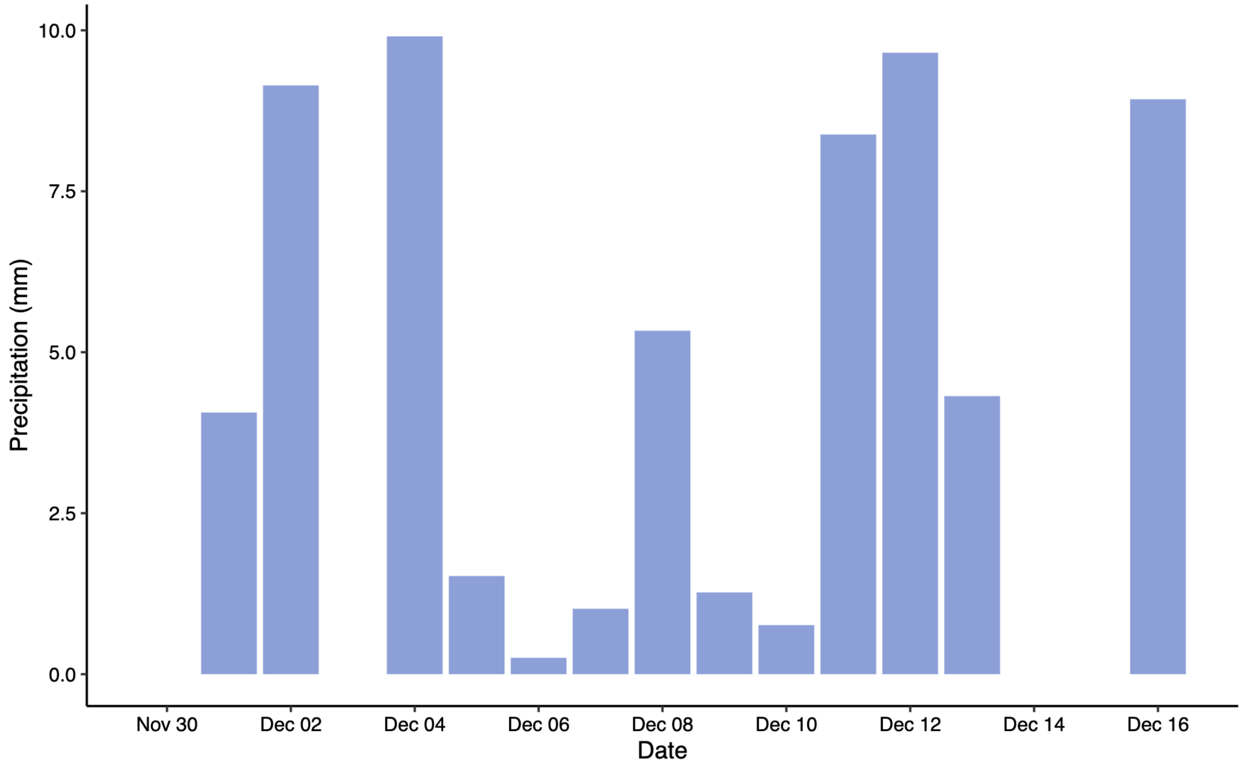

Figure S2. Distribution of detritus weight in milligrams (a) and water volume in milliliters (b) by bromeliad island.

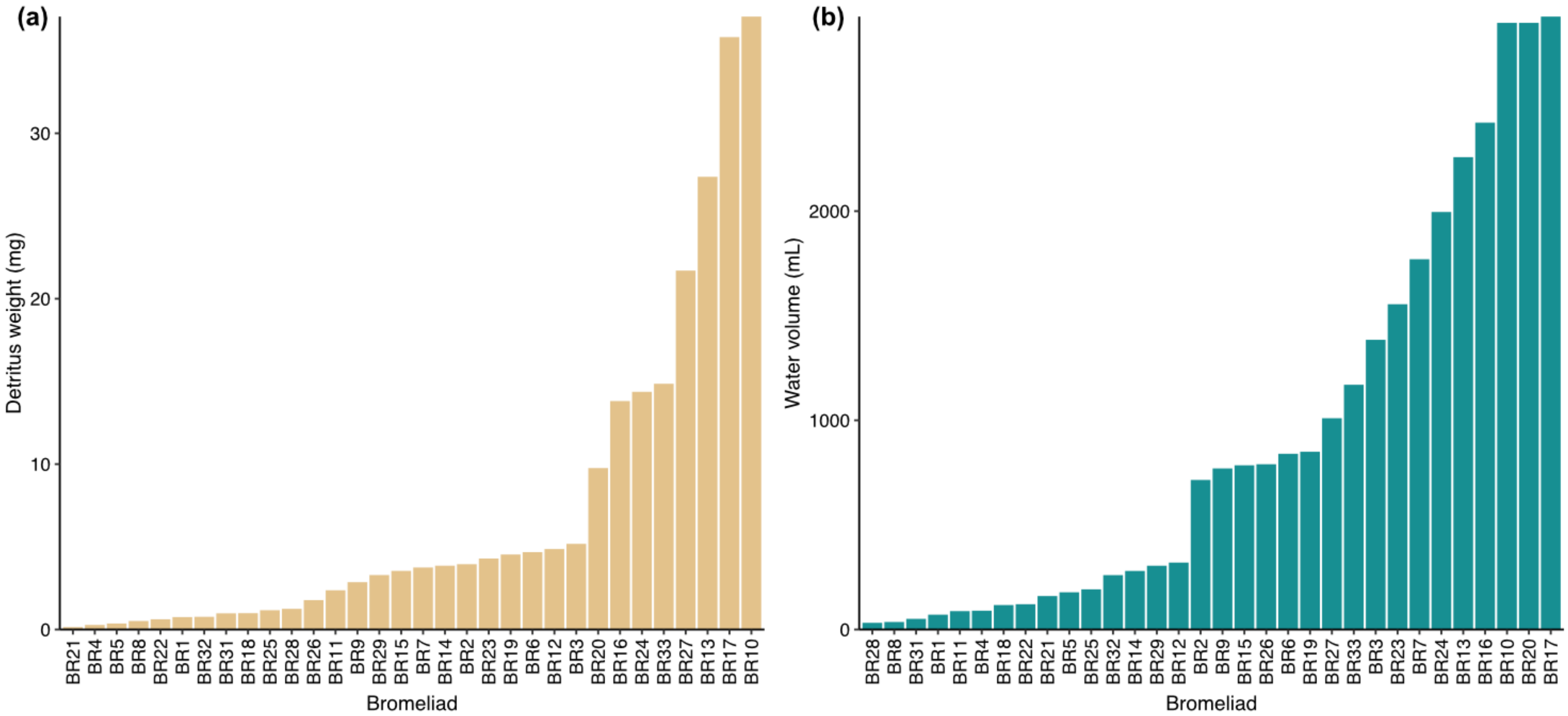

Figure S3. Log-transformed read distributions for fungi (a, b) and bacteria (c, d) used to determine cutoffs for quality control. Cutoffs by ASV are shown in blue, with a cutoff of three or fewer reads per ASV in fungi (a) and four or fewer reads in bacteria (c). Cutoffs by sample are shown in pink, with a cutoff of 300 or fewer reads per sample in fungi (b) and 2,900 or fewer reads in bacteria (d).

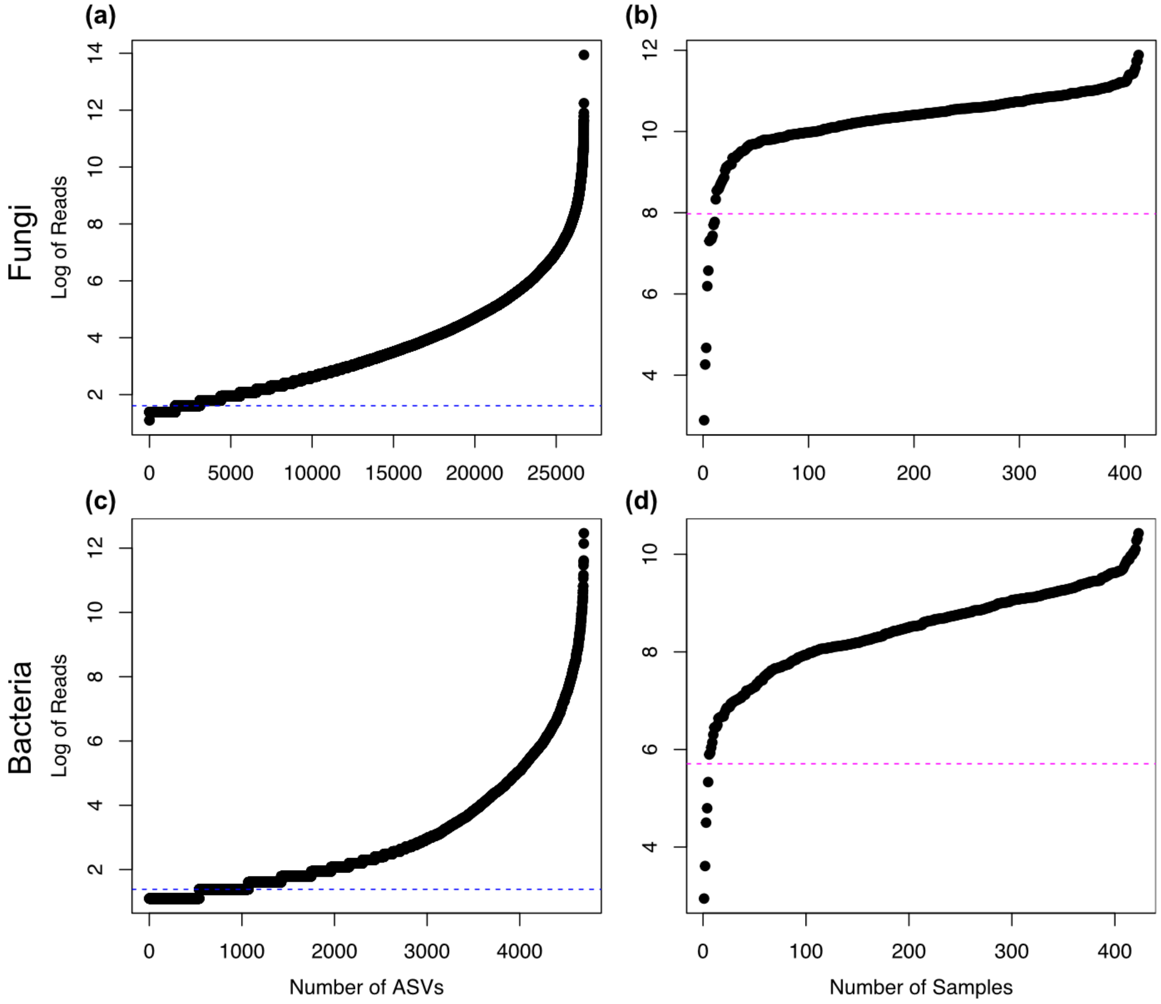

Figure S4. Accumulation curves of amplicon sequence variants (ASVs) across all fungal detritus samples. Solid lines indicate interpolation, dashed lines indicate extrapolation, and shaded areas represent 95% confidence intervals based on bootstrapping with 200 iterations.

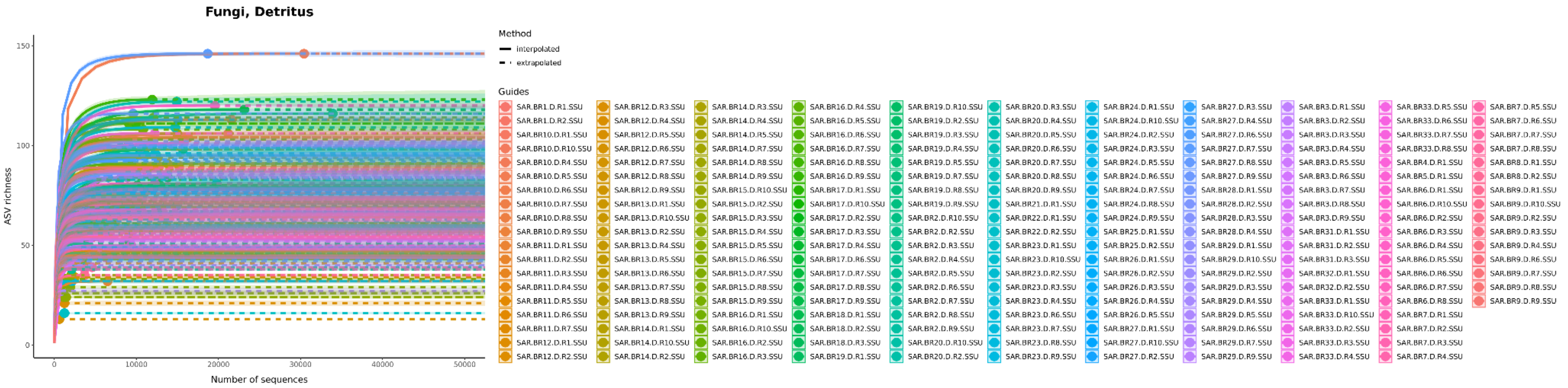

Figure S5. Accumulation curves of ASVs across all fungal water samples, as in Figure S4.

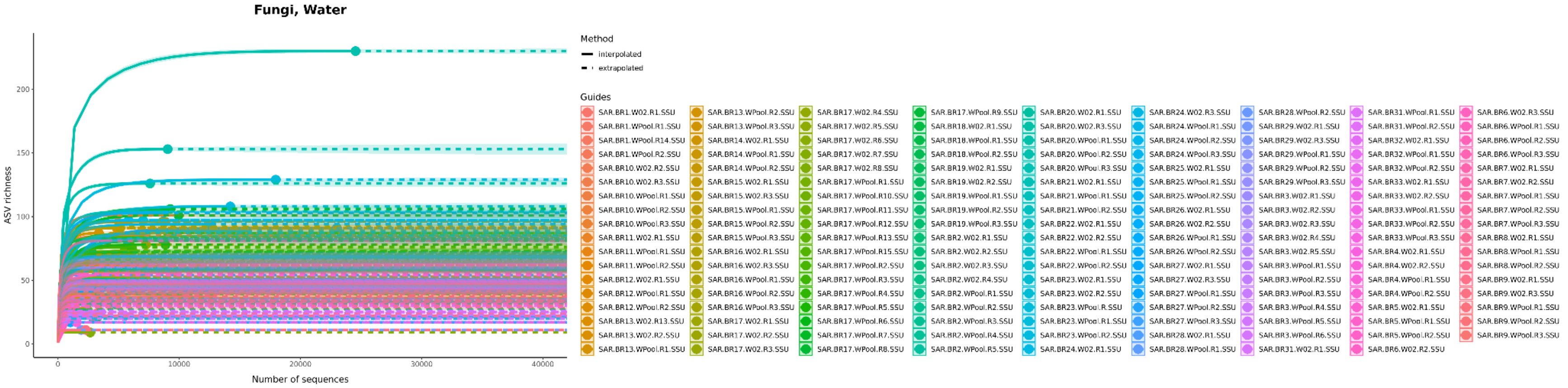

Figure S6. Accumulation curves of ASVs across all fungal invertebrate samples, as in Figure S4.

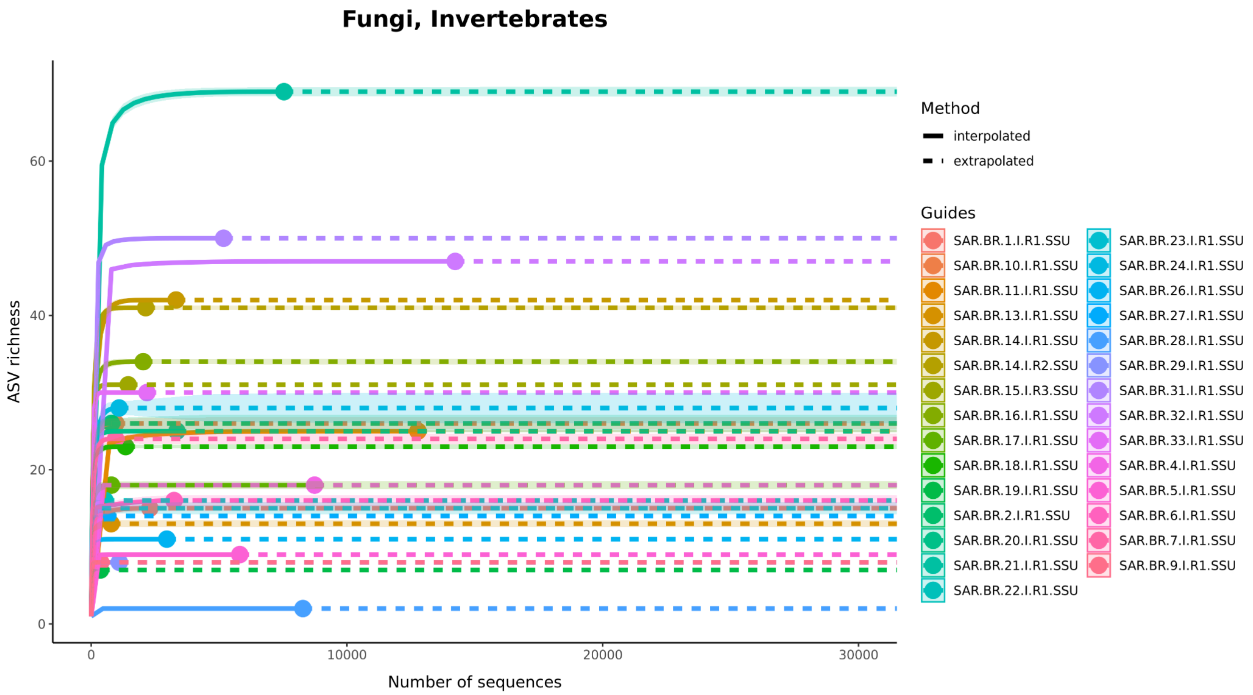

Figure S7. Accumulation curves of ASVs across all bacterial detritus samples, as in Figure S4.

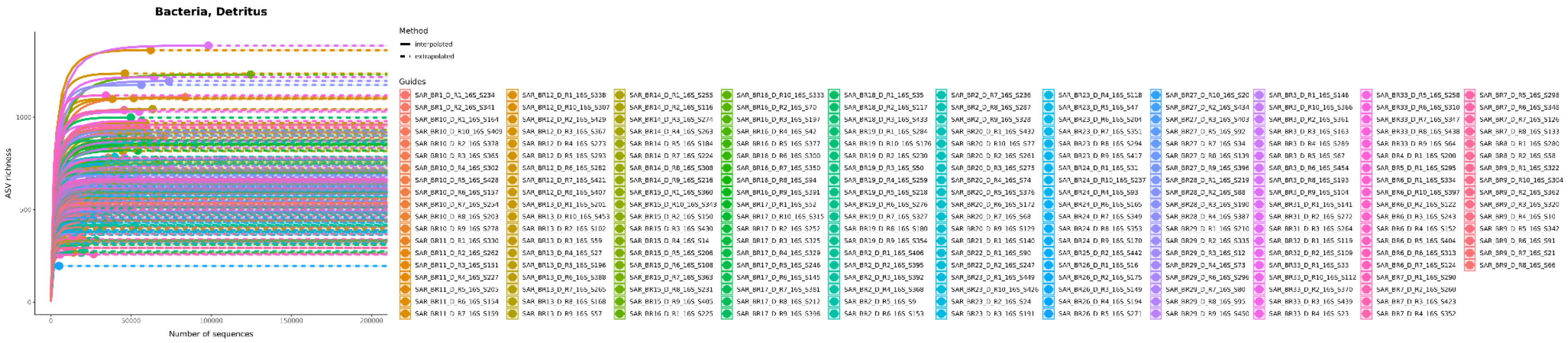

Figure S8. Accumulation curves of ASVs across all bacterial water samples, as in Figure S4.

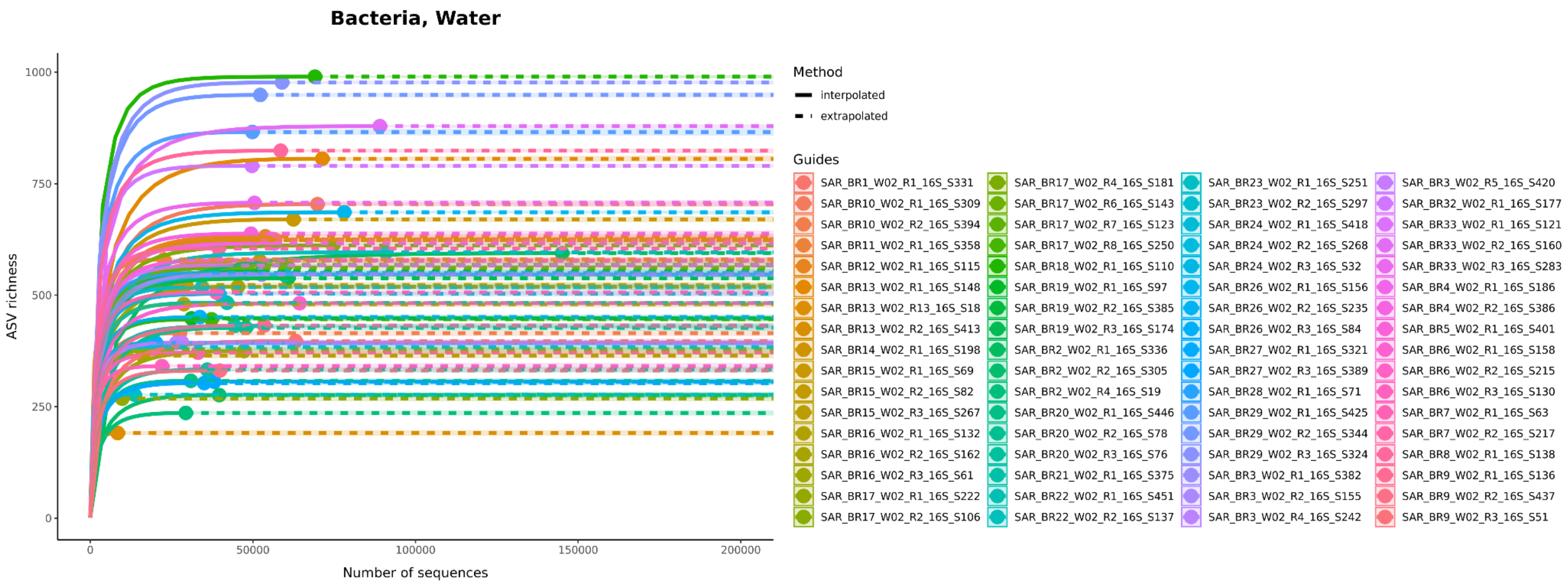

Figure S9. Accumulation curves of ASVs across all bacterial invertebrate samples, as in Figure S4.

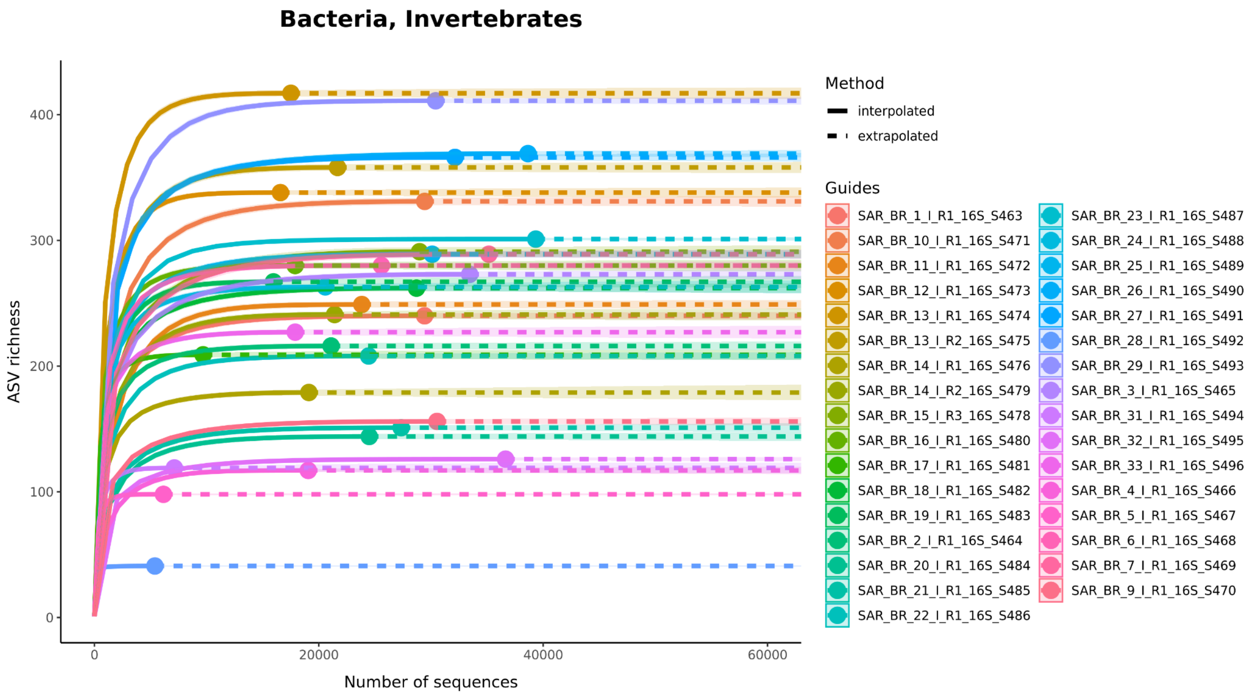

Figure S10. Relative abundance of fungal orders across bromeliad islands by compartment. All x-axes are ordered from left to right by smallest to largest island size.

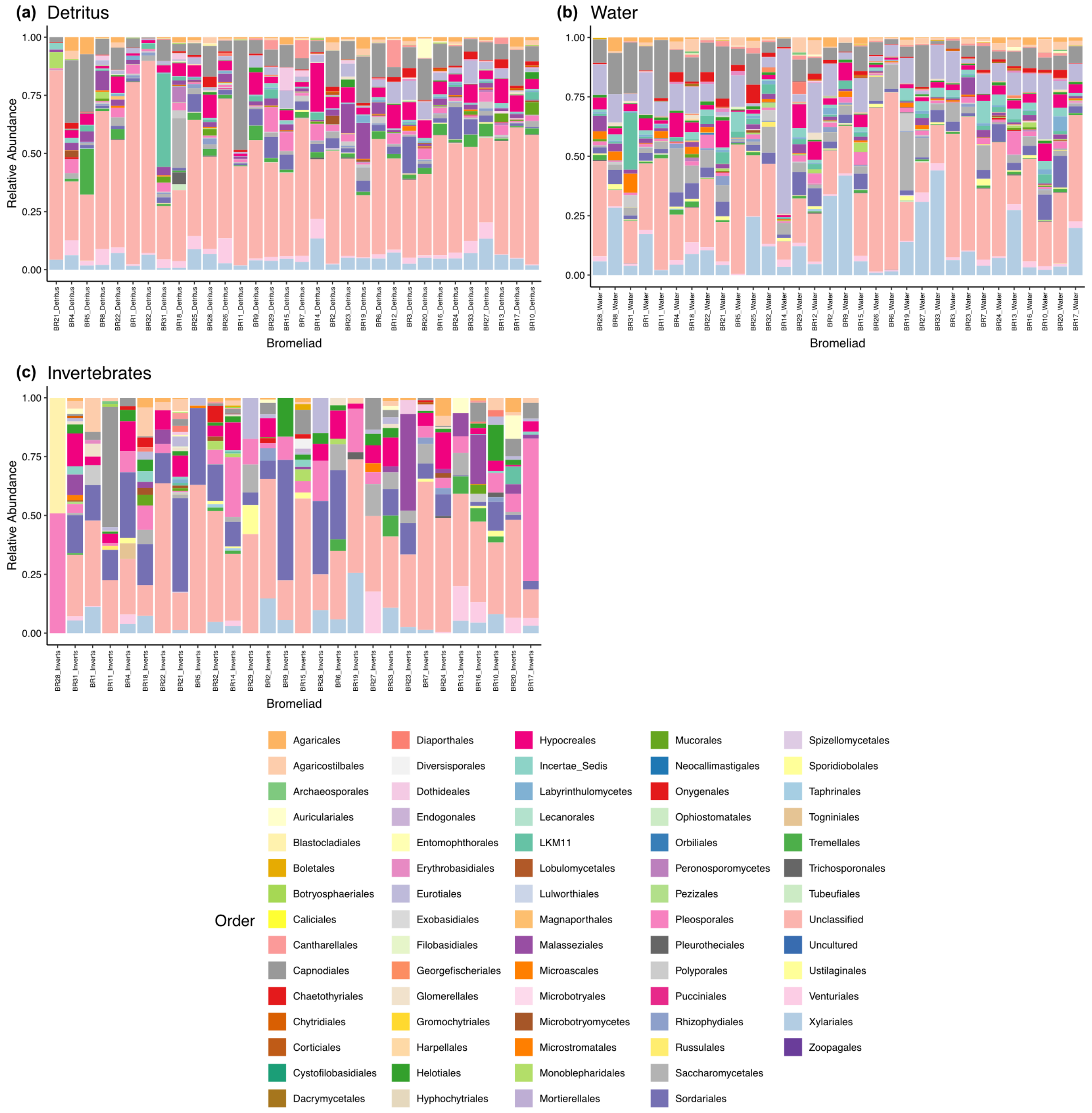

Figure S11. Relative abundance of bacterial classes across bromeliad islands, by compartment. All x-axes are ordered from left to right by smallest to largest island size.

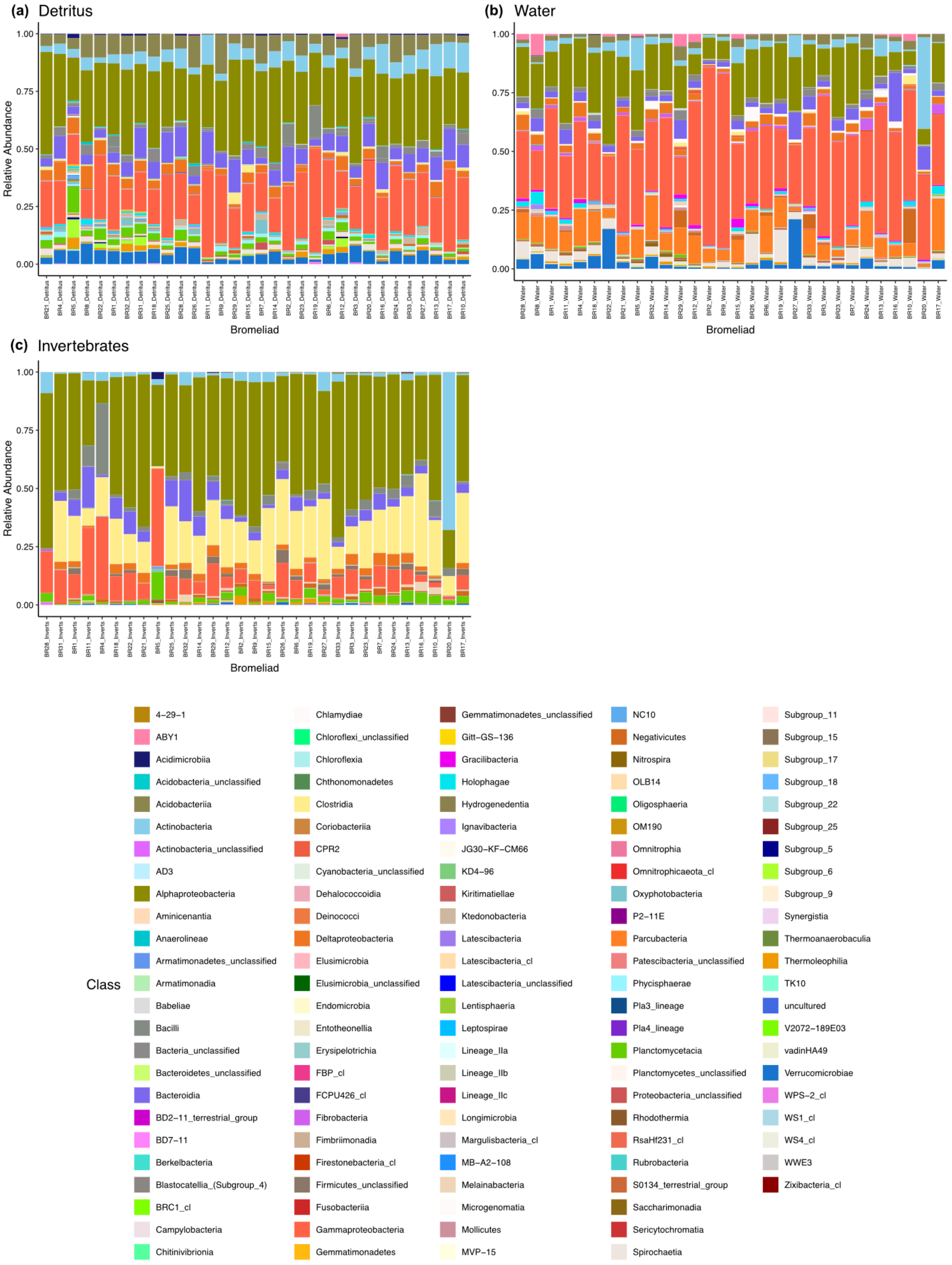

Figure S12. Plots show ordered 3 x ASV presence-absence matrices used to calculate nestedness (NODF) for (A) bacteria and (B) fungi across compartments (rows = water, detritus, invertebrates; columns = ASVs). Perfect nestedness would produce a triangular fill. Nestedness values (NODF) shown in Table S3.

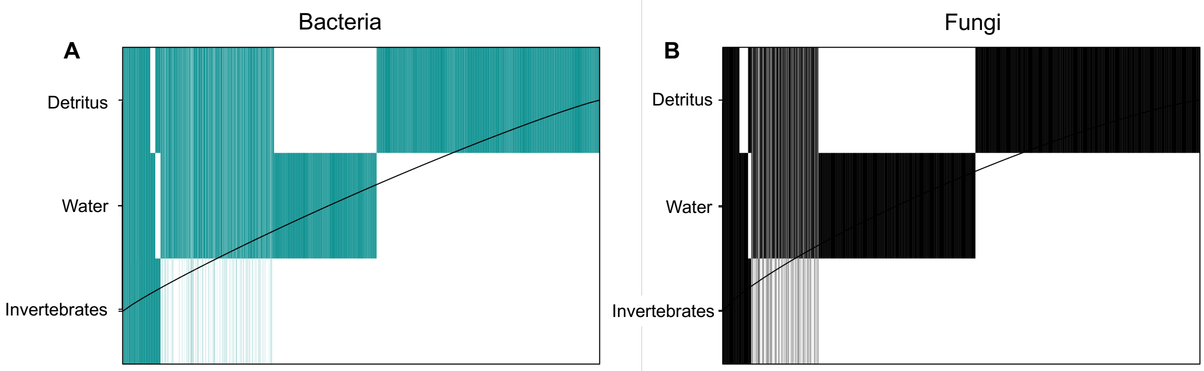

Figure S13. Microbial community evenness (Hill number $q = 1$) and bromeliad island size (log-transformed total detritus weight in mg or total water volume in mL) for fungi (A–D) and bacteria (E–H). $Q = 1$ was calculated for each sample (technical replicate) based on ASV abundances. Relationships are shown for detritus (A & E), water (B & F), and invertebrate samples (C–D & G–H), with each microbial locus analyzed independently. Regression lines are plotted only for significant relationships ($P < 0.05$). Regression slopes, intercepts, R^2 values, and P values are provided in Table S4.

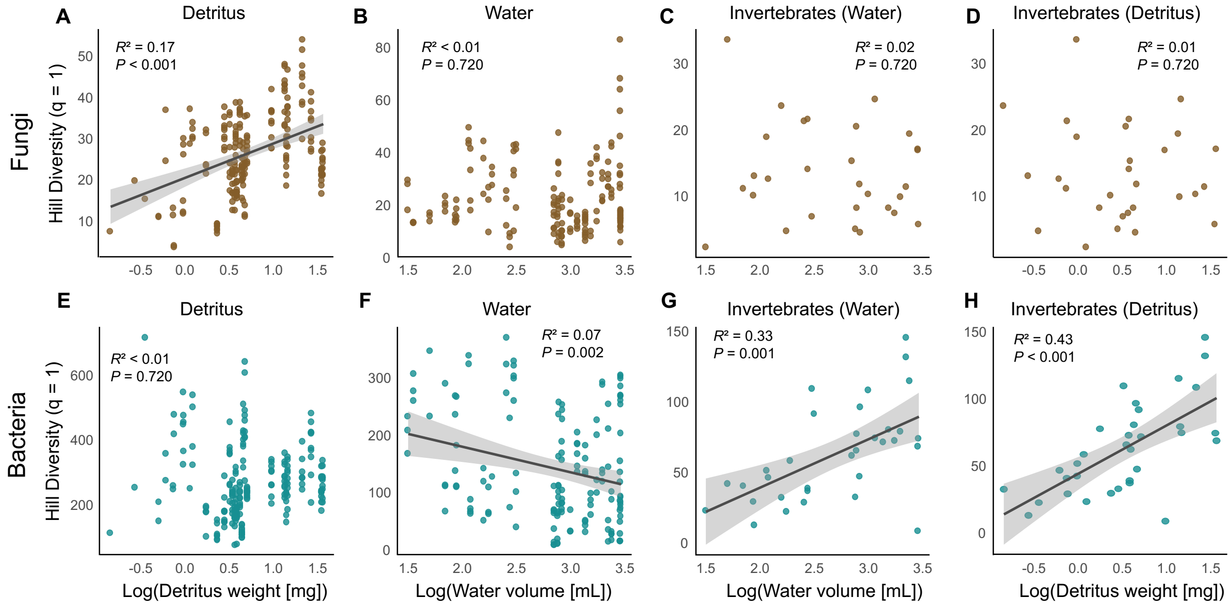

Figure S14. Slopes (z-scores) of SARs by fungi or bacteria and bromeliad compartment. Only z-scores for microbiomes with significant SARs are shown.

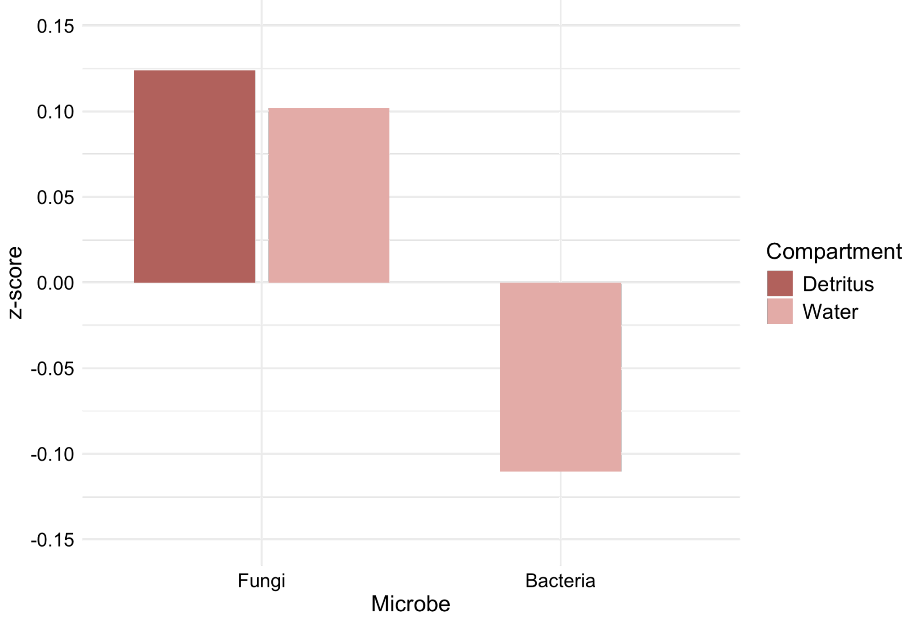

Figure S15. Relationship between (A) bromeliad island water pH and volume of water (as a representation of island size) and (B) the ratio of log-transformed detritus weight to log-transformed water volume and log-transformed water volume. pH significantly decreased ($P < 0.001$) as island size increased, where island size explained 34% of the variation in pH. The ratio of log-transformed island size significantly increased ($P < 0.001$) with log-transformed water volume.

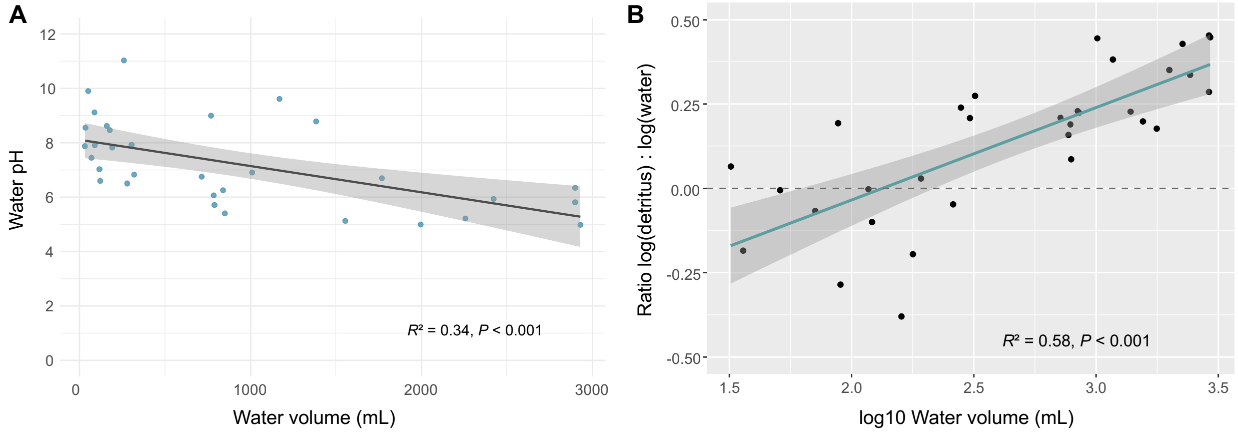

Figure S16. Relationships between dissolved oxygen (DO) concentration and microbial richness across bromeliads. Linear regressions indicated no significant relationship between DO and (A) fungal richness, or (B) bacterial richness.

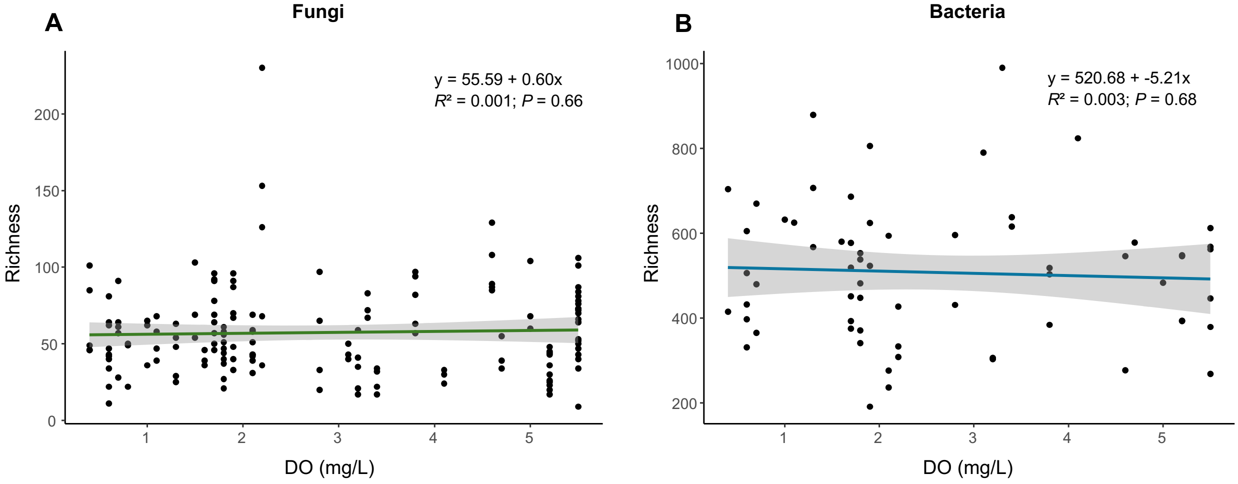

Figure S17. Relationship between bromeliad water volume and dissolved oxygen concentration. Linear regression revealed no significant association between water volume (island size) and dissolved oxygen ($R^2 = 0.01$, $P = 0.99$). Gray dashed line indicates estimated threshold of hypoxia.

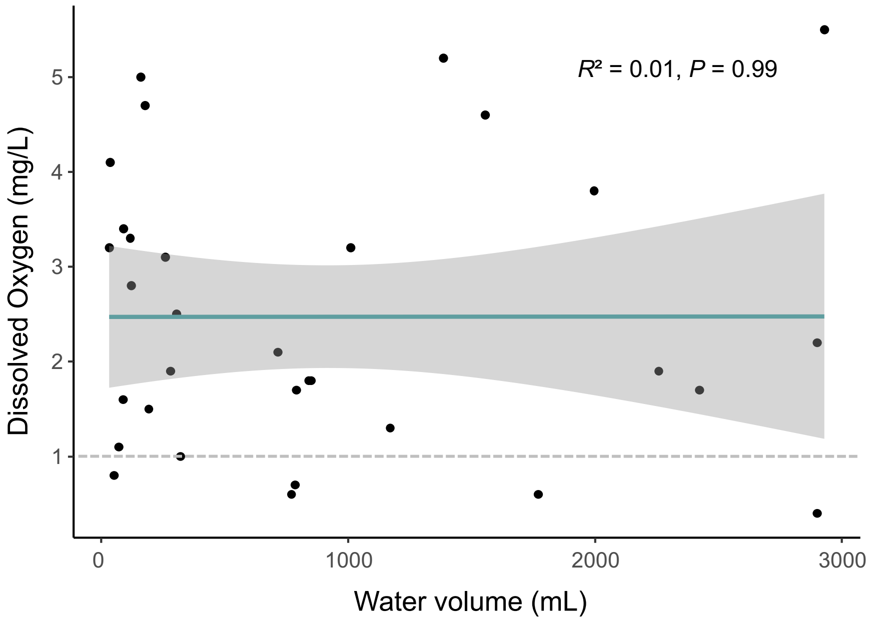

Supplementary Tables

Table S1. Number of samples (technical replicates) used for DNA extraction per bromeliad from 32 bromeliads, by compartment (water, invertebrates, detritus). The median number of technical replicates per bromeliad compartment were selected for DNA sequencing except in the case of the six bromeliads marked with an asterisk which were randomly selected to have all technical replicates sequenced. Some bromeliad islands had fewer than the median for water and detritus (3 per filter class for water, and 10 for detritus), in which case all replicates were sequenced, and one had no late-instar invertebrates.

Bromeliad	Water Tubes (Pooled)	Water Tubes (0.2 µm)	Invertebrate Tubes	Detritus Tubes
SAR_BR_1	2	1	1	2
*SAR_BR_2	5	4	1	10
*SAR_BR_3	6	5	1	10
SAR_BR_4	2	2	1	1
SAR_BR_5	2	1	1	1
SAR_BR_6	3	3	1	10
SAR_BR_7	3	2	1	8
SAR_BR_8	2	1	0	2
SAR_BR_9	3	3	1	10
SAR_BR_10	3	3	1	10
SAR_BR_11	2	1	1	7
SAR_BR_12	2	1	1	10
SAR_BR_13	3	3	1	10
SAR_BR_14	2	1	1	10
SAR_BR_15	3	3	2	10
SAR_BR_16	3	3	1	10
*SAR_BR_17	15	15	1	10

*SAR_BR_18	2	2	1	3
SAR_BR_19	3	3	1	10
SAR_BR_20	3	3	1	10
SAR_BR_21	2	1	1	1
SAR_BR_22	2	2	1	2
SAR_BR_23	3	2	1	10
SAR_BR_24	3	3	1	10
SAR_BR_25	2	1	1	2
SAR_BR_26	2	3	1	5
SAR_BR_27	3	3	1	10
SAR_BR_28	2	1	1	4
SAR_BR_29	3	3	1	10
*SAR_BR_31	1	1	1	3
*SAR_BR_32	1	1	1	2
SAR_BR_33	3	3	1	10

Table S2. Comparisons of microbial richness across bromeliad compartments and across loci. (a) Results of one-way and two-way ANOVAs testing the effects of bromeliad compartment (detritus, water, invertebrates) and microbial locus (fungi vs. bacteria) on observed microbial richness (ASV count). A global two-way ANOVA assessed main effects and interaction between compartment and locus, and separate one-way ANOVAs were performed for fungi and bacteria individually. All models were significant ($P < 0.001$). (b) Tukey HSD post hoc comparisons of microbial richness across bromeliad compartments for fungi and bacteria. Pairwise differences are shown with 95% confidence intervals and adjusted *P* values. Both fungi and bacteria exhibited the pattern Detritus > Water > Invertebrates. Statistically significant comparisons shown in bold ($p < 0.05$).

(a)

		**ANOVA**		
Model	Term	df	statistic	*P* value
Global (Fungi + Bacteria)	Compartment	2	89.8	**<0.001**
Global (Fungi + Bacteria)	Locus	1	292.63	**<0.001**
Global (Fungi + Bacteria)	Compartment x Locus	2	59.93	**<0.001**
Global (Fungi + Bacteria)	Residuals	179	NA	NA
Fungi only	Compartment	2	39.26	**<0.001**
Fungi only	Residuals	89	NA	NA
Bacteria only	Compartment	2	78.35	**<0.001**
Bacteria only	Residuals	90	NA	NA

(b)

Locus	Comparison	Mean difference	95% CI Upper	95% CI Lower	Adjusted *P* value
Fungi	Detritus x Water	-19.22	21.70	-60.14	0.504
Fungi	Detritus x Invertebrates	-146.38	-104.03	-188.74	**<0.001**

Fungi	Invertebrates x Water	-127.17	-84.81	-169.52	**<0.001**
Bacteria	Detritus x Water	-797.85	-548.70	-1046.99	**<0.001**
Bacteria	Detritus x Invertebrates	-1283.93	-1036.87	-1530.98	**<0.001**
Bacteria	Invertebrates x Water	-486.08	-235.00	-737.16	**<0.001**

Table S3. Global nestedness (NODF) values for fungi and bacteria across bromeliad compartments (water, detritus, invertebrates). Shown are observed NODF values, mean null expectations, standardized effect sizes (z-scores), and *P* values based on 1000 quasiswap randomizations (significant *P* values shown in bold). Richness (number of ASVs) in the total ASV pools are also provided.

Dataset	NODF Observed	NODF Null	NODF z-score	NODF *P* value	Total ASVs
Fungi	26.26	26.28	-4.73	**<0.001**	4024
Bacteria	41.81	41.90	-3.49	**<0.001**	23614

Table S4. Relative importance values of environmental and physical predictors in species-area models with detritus weight (a) or water volume (b) as a predictor. The response variable was always log-transformed ASV richness. Complexity refers to the number of leaves per bromeliad, and percent carbon and nitrogen were measured of the detritus. Dissolved oxygen, pH, temperature, and nitrate concentration were measured of the bromeliad water.

(a)

	Fungi		Bacteria	
	Invertebrates	Detritus	Invertebrates	Detritus

log(invertebrate_weight)	0.01	<0.01	0.01	<0.01
Diameter	0.01	0.04	0.01	0.01
Height	<0.01	0.03	0.01	0.03
Complexity	0.01	<0.01	<0.01	0.01
Invertebrate Number	<0.01	<0.01	0.04	0.03
Percent Carbon	0.05	<0.01	0.01	<0.01
Percent Nitrogen	0.01	0.01	0.02	0.01

(b)

	Fungi		Bacteria	
	Invertebrates	Water	Invertebrates	Water
log(invertebrate_weight)	0.02	0.03	<0.01	0.012
Diameter	0.02	0.01	0.04	0.070
Height	0.01	0.02	0.03	0.021
Complexity	0.03	0.01	<0.01	0.05
Invertebrate Number	0.03	0.01	0.01	0.01
Dissolved Oxygen	0.01	0.01	0.01	0.01
pH	0.20	0.08	0.06	0.07
Temperature	0.06	0.07	<0.01	0.01
Nitrate concentration	0.01	0.03	<0.01	<0.01

Table S5. Linear regression values for relationships between microbial diversity (Hill number $q = 1$) and log-transformed bromeliad size (detritus weight or water volume) across microbial loci (fungi and bacteria) and bromeliad compartments (detritus, water,

invertebrates). Slopes and intercepts were derived from linear models between Hill diversity and island size. P values were corrected with the Benjamini & Hochberg control of the false discovery rate (Benjamini & Hochberg 1995). Significant comparisons ($P < 0.05$) are in bold and are highlighted in Figure S12.

Locus	Compartment	R^2	Adjusted P value	Slope	Intercept
Fungi	Detritus	0.17	**<0.001**	8.34	19.86
Fungi	Water	<0.01	0.720	0.65	19.54
Fungi	Invertebrates (Water volume)	0.02	0.720	-1.53	17.26
Fungi	Invertebrates (Detritus weight)	0.01	0.720	-1.24	13.78
Bacteria	Detritus	<0.01	0.720	6.69	254.04
Bacteria	Water	0.07	**0.002**	-44.81	265.66
Bacteria	Invertebrates (Water volume)	0.33	**0.001**	34.26	-31.07
Bacteria	Invertebrates (Detritus weight)	0.43	**<0.001**	36.06	42.16

Table S6. Differences in bootstrapped slope distributions within bromeliad compartments (detritus, water, and invertebrates) between microbiome members (fungi and bacteria). Slopes were derived from the linear regression between log-transformed microbial richness and log-transformed bromeliad volume (detritus weight or water volume). Bootstrapped distributions were generated by randomly pairing richness and volume 1,000 times with replacement. Invertebrate microbial richness was regressed against both detritus weight and water volume, so the specific volume metric is in parentheses. Pairwise Welch's t-tests were performed and *P* values were corrected with the Benjamini & Hochberg control of the false discovery rate (Benjamini & Hochberg 1995). Significant comparisons *(P* ≤ 0.05) are in bold.

Fungal Compartment	Bacterial Compartment	*t*	df	Adjusted *P* value
Invertebrates (Water volume)	Invertebrates (Water volume)	10.73	1233.69	**<0.001**
Invertebrates (Detritus weight)	Invertebrates (Detritus weight)	-0.32	1187.33	0.750
Detritus	Detritus	71.19	1808.98	**<0.001**
Water	Water	169.03	1983.16	**<0.001**

Table S7. Differences in bootstrapped slope distributions of beta diversity over island size by bromeliad compartments (detritus, water, and invertebrates) within microbes (fungi and bacteria). Slopes were derived from the linear regression between Bray-Curtis dissimilarity values on community composition and bromeliad island size (detritus weight or water volume). Bootstrapped distributions were generated by randomly pairing community composition and weight or volume values 1,000 times with replacement. Invertebrate microbial beta diversity was regressed against both detritus weight and water volume, so the specific area metric is in parentheses. For invertebrate inhabiting fungi, only water volume was used, as the correlation between beta diversity and detritus weight was not significant (Figure 3). Pairwise Welch's t-tests were performed within microbes and *P* values were corrected with the Benjamini & Hochberg control of the false discovery rate (Benjamini & Hochberg 1995). All comparisons were significant ($P < 0.001$).

Locus	Compartment 1	Compartment 2	*t*	df	Adjusted *P* value	Slope 1	Intercept 1	Slope 2	Intercept 2
Fungi	Detritus	Invertebrates (Water volume)	125.18	1204.17	<0.001	0.09	0.73	0.05	0.89
	Detritus	Water	66.40	1923.18	<0.001	0.09	0.73	0.08	0.76
	Invertebrates (Water volume)	Water	-90.96	1301.06	<0.001	0.05	0.89	0.08	0.76
Bacteria	Detritus	Invertebrates (Detritus weight)	-45.58	1060.42	<0.001	0.09	0.75	0.12	0.68
	Detritus	Invertebrates (Water volume)	-55.99	1054.02	<0.001	0.09	0.75	0.12	0.68
	Detritus	Water	-199.24	1165.40	<0.001	0.09	0.75	0.16	0.67
	Invertebrates (Detritus weight)	Invertebrates (Water volume)	-9.42	1991.95	<0.001	0.12	0.68	0.12	0.68
	Invertebrates (Detritus weight)	Water	-67.89	1645.07	<0.001	0.12	0.68	0.16	0.67
	Invertebrates (Water volume)	Water	-53.91	1591.52	<0.001	0.12	0.68	0.16	0.67

Table S8. Differences in bootstrapped slope distributions of beta diversity over island size within bromeliad compartments (detritus, water, and invertebrates) between loci (fungi and bacteria). Slopes and bootstrapped distributions were derived as in Table S6. Only invertebrate slopes from regressions between community composition and water volume were performed (i.e., not detritus weight), as this correlation was not significant in invertebrate inhabiting fungi (Figure 3). Pairwise Welch's t-tests were performed as in Table S5. All comparisons were significant *(P* < 0.001).

Fungi	Bacteria	*t*	df	*Adjusted P* value	Slope (Fungi)	Intercept (Fungi)	Slope (Bact.)	Intercept (Bact.)
Invertebrates	Invertebrates	-121.24	1711.68	<0.001	0.05	0.89	0.13	0.68
Detritus	Detritus	-9.42	1901.90	<0.001	0.09	0.73	0.09	0.75
Water	Water	-228.41	1379.00	<0.001	0.08	0.76	0.16	0.67

Table S9. Summary of abundance-based Raup-Crick (RC_{Bray}) results for fungal and bacterial communities across bromeliad compartments (water, detritus, invertebrates). RC_{Bray} values were categorized following Stegen et al (2013) into homogenizing dispersal ($RC_{Bray} < -0.95$), drift ($-0.95 < RC_{Bray} < 0.95$), and dispersal limitation + drift or environmental selection ($RC_{Bray} > 0.95$). Frequency values indicate the proportion of pairwise community comparisons falling into each category.

Raup Crick (Bray)			
Locus	Compartment	Community assembly process	Frequency
Fungi	Detritus	Homogenizing dispersal	0.00
Fungi	Detritus	Drift	<0.01
Fungi	Detritus	Dispersal limitation + drift or environmental selection	>0.99
Fungi	Water	Homogenizing dispersal	0.00
Fungi	Water	Drift	<0.01
Fungi	Water	Dispersal limitation + drift or environmental selection	>0.99
Fungi	Inverts	Homogenizing dispersal	0.00
Fungi	Inverts	Drift	0.00
Fungi	Inverts	Dispersal limitation + drift or environmental selection	1.00

Bacteria	Detritus	Homogenizing dispersal	<0.01
Bacteria	Detritus	Drift	<0.01
Bacteria	Detritus	Dispersal limitation + drift or environmental selection	>0.99
Bacteria	Water	Homogenizing dispersal	<0.01
Bacteria	Water	Drift	<0.01
Bacteria	Water	Dispersal limitation + drift or environmental selection	>0.99
Bacteria	Inverts	Homogenizing dispersal	0.00
Bacteria	Inverts	Drift	0.00
Bacteria	Inverts	Dispersal limitation + drift or environmental selection	1.00

Supplementary References

Benjamini, Y. & Hochberg, Y. (1995). Controlling the false discovery rate: a practical and powerful approach to multiple testing. *Journal of the Royal statistical society: series B (Methodological)*, 57, 289–300.
